# Environmental Risks of Pesticide Residues in the Lake Tana Sub-basin, Ethiopia: A Review

**DOI:** 10.1007/s00267-026-02399-z

**Published:** 2026-02-18

**Authors:** Banchiamlak Getnet Admasu, Kaisheng Yao, Goraw Goshu Yemer, Paul J. Van den Brink

**Affiliations:** 1 Aquatic Ecology and Water Quality Management Group, Wageningen University, Wageningen, the Netherlands; 2 College of Agriculture and Environmental Sciences, Bahir Dar University, Bahir Dar, Ethiopia; 3 Blue Nile Water Institute, Bahir Dar University, Bahir Dar, Ethiopia

## Abstract

Lake Tana, Ethiopia’s largest natural lake and the headwater of the Blue Nile, provides critical ecosystem services and socio-economic benefits. However, rapid agricultural expansion in the Lake Tana sub-basin has led to increased pesticide use, which raises serious environmental concerns. This systematic review synthesi zes information on agricultural pesticide use, their residues in fish, water, and sediments, and the associated ecological and human health risks. A total of 66 active ingredients were identified across 13 districts, with the highest pesticide use reported in Libokemkem, Fogera, and Gondar Zuria. Pesticide use was dominated by insecticides, including several WHO-classified hazardous compounds. Risk Quotients derived from fish body residues identified imidacloprid, oxamyl, and flazasulfuron as priority pesticides posing high to very high risks to *Labeobarbus* spp. In Lake Tana, legacy organochlorine pesticides (endosulfan, lindane, endrin, DDT, and dieldrin) posed high to very high risks to *Oreochromis niloticus*. Human health risk assessment based on fish consumption indicated negligible non-carcinogenic risks from both current-use and legacy pesticides. Potentially Affected Fraction (PAF) and multi-substance Potentially Affected Fraction (msPAF) analyses indicated severe ecological risks posed by the measured water concentrations across the Lake Tana sub-basin. Sediment-bound pesticides also posed consistently high ecological risks, particularly for persistent organochlorines (lindane, endrin, and dieldrin). Overall, these findings advocate the urgent need for improved pesticide regulation, better management of legacy contaminants, and the promotion of sustainable agricultural practices to protect the Lake Tana sub-basin and its vital ecosystem services.

## Introduction

Ecosystems can significantly contribute to people’s quality of life and well-being, with the magnitude and nature of these benefits shaped by cultural and socioeconomic contexts. Lake Tana is one of Ethiopia’s most important natural assets, providing substantial ecological and socioeconomic benefits. It is the largest natural lake in the country and serves as the headwater of the Blue Nile Basin (Belete 2014; Asitatikie and Nigussie, 2021). Lake Tana supports a rich diversity of flora and fauna, including several endemic fish species that are critical to local and regional biodiversity (Mengistu et al. 2017). The extensive wetlands of Lake Tana play a great role in regulating water flow, maintaining water quality, and providing essential breeding and nursery habitats for bird and fish species, making the basin a key hotspot for aquatic and avian biodiversity (Anteneh et al. 2012; Aynalem and Mengistu, 2017). The wetland hydrology indicator map shows that wetlands in the Lake Tana sub-basin are widespread, with permanently inundated areas covering approximately 591,312 ha, increasing to 607,053 ha when temporarily inundated wetlands are included (Wondim et al. 2025). Through nutrient retention and sediment trapping, these wetlands support ecosystem functioning and contribute significantly to biodiversity conservation. Owing to its unique ecological value, Lake Tana has been designated as a UNESCO Biosphere Reserve (Worku 2017). Beyond its ecological importance, Lake Tana supports the livelihoods of millions of people through fisheries, agriculture, and tourism, linking its ecological health directly to the social and economic well-being of surrounding communities (Gordon et al. 2007). The ecosystem services provided by the lake and its wetlands are essential not only for local and regional environmental balance but also for sustaining downstream ecosystems and agricultural systems in Sudan and Egypt (Goshu and Aynalem 2017).

The economy of the Lake Tana sub-basin is primarily driven by agriculture, which offers substantial potential for irrigation development (Berihun 2017). In addition to agriculture, the fisheries sector plays a vital role in supporting livelihoods, ensuring food security, and generating regional income. Currently, an estimated 3514 active fishers operate in the sub- basin; assuming an average of six dependents per household, this translates to more than 21,000 individuals directly benefiting from fishing activities. When fish processors, traders, gillnet makers, and boat builders are included, the total number of primary beneficiaries exceeds 40,000 (Mengistu et al. 2017).

To make use of the agricultural potential, the government has proposed several irrigation schemes, including Megech‑Seraba, Tana Asrate, Tana Mekonta, Tana Wenjeta, Tana Zegie, Shina, Bebekis, Kuhar‑Michael, and Koga (Fog. 2). Table S1 summarizes the design and actual irrigated land, operational status, and locations of these schemes. Among these, the Koga scheme is fully operational, irrigating approximately 6,508 ha, while Megech‑Seraba is partially operational with 566 ha currently under irrigation (Taye et al. 2021). Other schemes, such as Tana Asrate, Tana Mekonta, and Tana Zegie, are small‑scale and locally operated (Mequanent et al. 2019), whereas Shina and Bebekis operate with variable small-scale pump or diversion systems (Abera et al. 2019). The operational status and scale of these irrigation schemes influence horticultural crop production and have contributed to increased pesticide use for pest management and vector control in irrigated areas of the Lake Tana sub-basin (Merkuz 2017). The introduction of rice cultivation as a major crop in the Fogera and Dembia floodplains has also contributed to increased pesticide use (Atnafu et al. 2011; Goshu and Aynalem, 2017). These pesticides may pose significant environmental and public health risks within the sub-basin (Merkuz 2017). Many farming communities lack adequate information about the hazards associated with these pesticides, leading to their misuse (Merkuz 2017; Agmas and Adugna 2020; Abaineh et al. 2024).

Several studies have documented pesticide misuse and contamination in fish, water, and sediment in the Lake Tana sub-basin, highlighting potential ecological risks (Abera et al. 2022; Zelalem et al. 2023). However, the existing evidence is fragmented, and a comprehensive synthesis of pesticide occurrence and associated risks is lacking. In this context, this systematic review compiles and evaluates the available literature on agricultural pesticide residues in fish, water, and sediments in the Lake Tana sub-basin. The review further assesses their ecological and human health risks using established risk assessment frameworks, providing an integrated understanding of pesticide exposure and its implications for aquatic ecosystems and local communities. This review focuses exclusively on pesticides used for agricultural purposes, as agriculture represents the primary and most widespread source of pesticide inputs to the sub-basin. Agricultural pesticides enter aquatic ecosystems mainly through surface runoff, irrigation return flows, and sediment mobilization, making them particularly relevant for ecological and human health risk assessment. Pesticides used for household, public health, or industrial applications were excluded because their sources, exposure pathways, and regulatory frameworks differ substantially, and existing monitoring data do not allow reliable attribution of aquatic contamination to these uses.

This review is intended to support policymakers and regulatory authorities by providing an integrated assessment of pesticide occurrence and associated risks, assisting environmental and public health practitioners in identifying priority contaminants and exposure pathways, and informing water and irrigation managers about the implications of agricultural intensification for aquatic ecosystems. In addition, it emphasizes key data gaps and research needs for scientists and monitoring agencies, thereby contributing to evidence-based decision-making and the sustainable management of the Lake Tana sub-basin.

## Methods

### Systematic Review

**Fig. 1:**
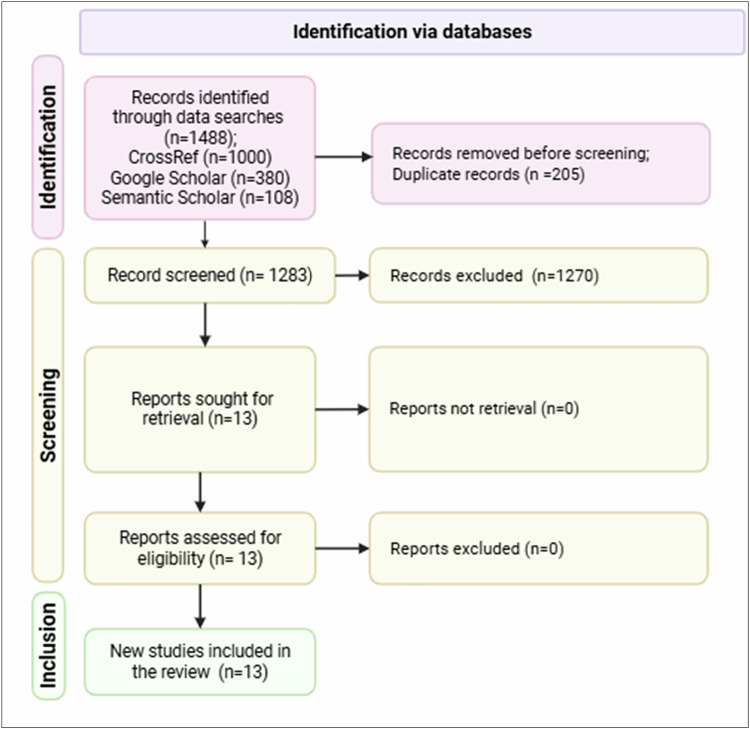
PRISMA flow diagram for the systematic review process

A systematic review was conducted to identify studies reporting the types, uses, and environmental residues in fish, water, and sediments within the Lake Tana sub-basin. The review covered peer-reviewed and gray literature published between 2015 and 2024. Literature searches were performed in three scientific databases/search engines: CrossRef, Google Scholar and Semantic Scholar using the Publish or Perish software (https://harzing.com/resources/publish-or-perish). Search terms consisted of combinations of pesticides, herbicides, insecticides, fungicides, pesticide residues, and risk assessment. The study selection process followed the PRISMA 2020 guidelines and is summarized in Fig. 1. A total of 1488 records were identified across the three scientific databases. After removal of duplicate records (*n* = 205), the titles and abstracts of 1283 records were screened for relevance. Thirteen (*n* = 13) studies were subsequently retrieved and assessed in full for eligibility, all of which met the inclusion criteria and were included in the review.

Studies were included if they (i) were conducted within the Lake Tana sub-basin, (ii) reported concentrations of agricultural pesticides in water, sediment, or fish, and (iii) were published between 2015 and 2024. Eligible studies provided sufficient quantitative or qualitative data to support ecological or human health risk assessment and consisted of peer-reviewed articles or accessible gray literature (e.g., conference proceedings and technical manuals) published in English. Studies conducted outside the Lake Tana sub-basin, opinion papers, studies lacking primary data, or those unrelated to pesticide exposure were excluded. Articles published in local languages were not considered due to accessibility limitations.

**Fig. 2:**
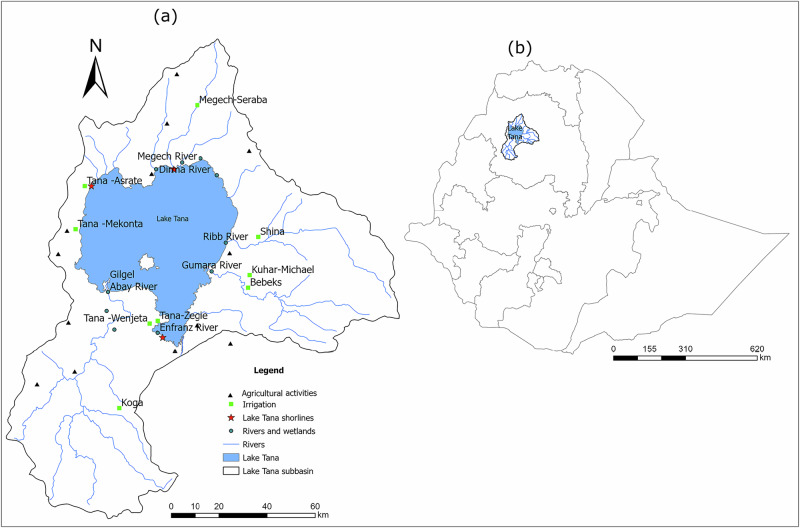
Map of the Lake Tana sub-basin showing (**a**) its location within Ethiopia and **b** the distribution of study sites, including areas of different agricultural activities, irrigation schemes, Lake Tana shorelines, rivers, and wetlands where pesticide-related studies were conducted. The sampling sites are derived from both environmental monitoring studies reporting measured pesticide residues (Abera et al. 2022; Agmas and Adugna, 2022; Tibebe et al. 2022; Zelalem et al. 2023; Abaineh et al. 2024) and survey-based studies documenting pesticide uses (Begna, 2015; Agmas and Adugna, 2020; Asmare et al. 2022; Sishu et al. 2022; Abaineh et al. 2023; Abera et al. 2022 (both monitoring and survey); Alebachew et al. 2023; Tassew et al. 2023)

From the 13 eligible studies, data were extracted on pesticide types, environmental concentrations, and associated ecological and human health risks. In addition, qualitative information on factors contributing to pesticide misuse and overuse, such as socio-economic constraints, limited farmer education, and regulatory challenges, was collated. Quantitative data on potential ecological and human health risks were evaluated by comparing reported concentrations with relevant guideline thresholds. Most studies included in this review were based on limited sampling locations or focused on a single fish species per site, which may not fully capture the spatial variability of pesticide contamination across the Lake Tana sub-basin (Fig. 2). These limitations should be considered when extrapolating findings to broader spatial scales, and caution is therefore warranted when interpreting basin-wide risk estimates.

### Fish Risk Assessment

To evaluate the risk to fish, the risk quotient (RQ) methodology was applied. This method calculates the RQ by dividing the Measured Environmental Concentration in biota as expressed as body residue (MECbr) by the Predicted No-Effect Concentration in biota (PNECbr). MECbr values (in µg kg^–1^ wet weight) were derived from pesticide concentrations in fish as reported in the reviewed studies, while PNECbr values were obtained from the NORMAN Ecotoxicology Database (https://www.norman-network.com/nds/ecotox/). The Risk Quotient (RQ) for pesticides in fish was calculated using the following formula:

1 $${\rm{RQ}}=\frac{{\rm{MECbr}}}{{\rm{PNECbr}}}$$

Where;

MECbr (µg kg^−1^ wet weight) represents measured pesticide concentrations in fish tissues, extracted from reviewed studies. PNECbr (µg kg^−1^wet weight) values were obtained from the NORMAN Ecotoxicology database (https://www.norman-network.com/nds/ecotox/). which compiles standardized toxicity data for fish and other aquatic organisms. Risk categories were defined following Merga et al. (2021): RQ < 1 indicates no risk, 1 ≤ RQ < 10 indicates low risk, and 10 ≤ RQ < 100 indicates high risk.

This assessment was limited to three fish species, *Labeobarbus megastoma, Labeobarbus tsanensis*, and *Oreochromis niloticus*, as these were the only species for which sufficient measured pesticide concentration data were available in Lake Tana. These species represent key trophic positions (piscivorous, insectivorous, and herbivorous) (Zelalem et al. 2023), but may not capture risks to more sensitive species, early life stages, or other trophic levels. Consequently, the results provide an estimate of fish-level risks. Future studies should expand monitoring efforts to include additional species, age classes, and invertebrate taxa to enable a more comprehensive assessment of ecosystem-level risks.

### Human Health Risk Assessments

Human health risks associated with pesticide residues in fish were assessed in accordance with United States Environmental Protection Agency (USEPA) guidelines, considering both non-carcinogenic (chronic systemic) and carcinogenic (cancer) risks from dietary exposure (https://www.epa.gov/risk/guidelines-carcinogen-risk-assessment). The Estimated Daily Intake (EDI) carcinogenic and non-carcinogenic estimates were used to comprehensively assess health risks. The Estimated Daily Intake (EDI) of each pesticide in the various fish was calculated using the following equation:

2 $${\rm{EDI}}({\rm{mg}}/{\rm{kg}}/{\rm{day}})=\frac{{\rm{MEC\; X\; IR}}}{{\rm{BW}}}$$

Where: MEC mean measured concentration of the pesticide in fish muscle (mg/kg wet weight), obtained from previous studies, while IR is the fish ingestion rate (kg/person/day). A standard consumption rate of 0.03 kg/person/day (Yohannes et al. 2014) was used. BW is the average adult body weight (kg), assumed to be 60 kg (Teklu et al. 2015).

Potential non-cancer effect was calculated using the Target Hazard Quotient (THQ) equation:

3 $${\rm{THQ}}=\frac{{\rm{EDI}}}{{\rm{RfD}}}$$

Where: RfD is the oral reference dose (mg/kg-bw/day), representing a daily exposure level unlikely to cause adverse health effects over a lifetime (USEPA IRIS) (https://www.epa.gov/iris). To account for exposure to multiple pesticides, the Hazard Index (HI) was calculated using the following equation:

4 $$\mathrm{HI}={\sum }_{{\rm{i}}=1}^{{\rm{n}}}\mathrm{THQi}$$

HI < 1 indicates that the overall non-carcinogenic risk is acceptable; HI ≥ 1 suggests a potential health concern (Kone et al. 2023).

Only the pesticides for which cancer slope factors (CSFs) are available in authoritative databases, such as lindane, DDT, and dieldrin, were considered for the calculation of their carcinogenic risks in this review. The lifetime cancer risk for pesticides classified as carcinogens was calculated using the Incremental Lifetime Cancer Risk (ILCR) equation:

5 $${\rm{ILCR}}={\rm{EDI}}\times {\rm{CSF}}$$

Where: CSF is the cancer slope factor ((mg/kg-bw /day)⁻¹), representing the increased lifetime cancer probability per unit of daily exposure (Li 2018). The cumulative carcinogenic risk was calculated as;

6 $$\mathrm{Cummulative\; ILCR}={\sum }_{{\rm{i}}=1}^{{\rm{n}}}\mathrm{ILCRi}$$

With ILCR ≤ 0.000001 representing a negligible risk, ILCR > 0.000001 to ≤ 0.0001 an acceptable to tolerable risk, and ILCR > 0.0001, a potentially unacceptable risk (Badibostan et al. 2019).

### Biodiversity Risk Assessments

To assess the ecological risks posed by pesticide residues in water to aquatic biodiversity, probabilistic risk assessment methods, the Potentially Affected Fraction (PAF) and the multi-substance Potentially Affected Fraction (msPAF), were employed. These metrics can serve as biodiversity impact metrics by quantifying the proportion of aquatic species potentially impacted by the individually measured pesticide concentrations and their mixture (Posthuma and de Zwart, 2006https://tactiq.io/r/transcribing). The PAF and msPAF were calculated based on acute and chronic toxicity data using the PAF calculator developed by “Kennis Impuls Water Kwaliteit” (https://www.sleutelfactortoxiciteit.nl/key-factor-toxicity-introduction). The acute and chronic PAF values indicate which fractions of the species exhibit acute (assessed using EC50s) or chronic (assessed using NOECs) toxicity at concentrations lower than the exposure concentration, calculated using the Species Sensitivity Distribution (SSD) concept. From these PAF values, the msPAF values were calculated for the measured mixtures measured in the same sample (Posthuma et al. 2019).

To assess the ecological risks posed by pesticide residues in sediments, a risk quotient (RQ) approach was applied using the ratio of measured environmental concentrations in sediment (MEC sediment) to the predicted no-effect concentration for sediment (PNEC sediment). Measured concentrations (µg kg⁻¹ dry weight) were obtained from published sediment monitoring studies, while PNEC values were sourced from the NORMAN Ecotoxicology database (https://www.normannetwork.com/nds/ecotox/lowestPnecsIndex.php). The RQ was calculated using the following equation:

7 $${\rm{RQ}}=\frac{{\rm{MEC\; sediment}}}{{\rm{PNEC\; sediment}}}$$

Where;

MEC represents the measured pesticide concentration in the sediment. PNEC values were obtained from the NORMAN Ecotoxicology database (https://www.normannetwork.com/nds/ecotox/) which compiles standardized toxicity data for aquatic organisms in the sediment. Risk levels were classified according to Nie et al. (2015): RQ < 0.1 indicates no risk; 0.1 ≤ RQ < 1, a low risk; 1 ≤ RQ < 10, a medium risk; and RQ ≥ 10, a high risk.

## Results and Discussion

### Pesticide Types

Table S2 and Fig. S1 show the various types of pesticides used across 13 districts (Fig. 2). Among these, Libokemkem, Fogera, and Gondar Zuria exhibit the highest number of active ingredients ( > 30), while Mecha and Dembia also show high levels (30). Alefa, Robit, North Achefer and South Achefer stand out with the lowest numbers (10-13). Overall, farmers across these districts applied 66 different active ingredients (Table S2), with high numbers particularly linked to the recent surge in small-scale irrigation projects in the area (Worqlul et al. 2015). The Fogera district was identified by Taye et al. (2021) as having the largest irrigated area and ranking second in the number of pesticides used, after Libokemeken (Fig. S1). Some of the active ingredients are classified as hazardous by the WHO (2020). For example, highly hazardous (Class Ib) active ingredients include methomyl, terbufos, 2,4-D, endosulfan, and dichlorodiphenyltrichloroethane (DDT). Moderately hazardous (Class II) active ingredients comprise chlorpyrifos, dimethoate, cypermethrin, deltamethrin, profenofos, propiconazole, mancozeb, bifenthrin, and imidacloprid. Additionally, glyphosate and malathion are categorised as slightly hazardous (Class III) (Table S2).

Based on the number of active ingredients, insecticides outnumbered those of herbicides, fungicides, and rodenticides (Fig. S1), indicating an extensive reliance on insecticides to manage common pest incidents. The predominant insects targeted in the area include aphids, African bollworm caterpillars, thrips, termites, spider mites, stock borers, fruit borers, and soil-borne insects affecting crops like cabbage, onion, tomato, pepper, maize, chickpea, grass pea, wheat, teff, fruits, and khat (Agmas and Adugna, 2020). Agmas and Adugna (2020) observed that farmers commonly apply multiple pesticide types, with insecticides being the most frequently used, followed by herbicides and fungicides. Similarly, the study by Abera et al. (2022) reported that most of the pesticides identified in the Lake Tana basin were insecticides. Additionally, Sishu et al. (2022) reported that all identified pesticides in their Lake Tana study were insecticides used specifically to control aphids. Herbicides are the second-most-used pesticides, particularly in the Fogera district, where they play a key role in rice production. Farmers in this region face significant weed challenges, prompting widespread herbicide use to protect their crops (Chauhan and Johnson 2010; Assefa et al. 2011; Jaipaul et al. 2014).

In this study, fungicides and rodenticides were found to have a lower utilization compared to insecticides and herbicides. This could be attributed to the inherent resistance of the predominant crops to fungal diseases, reducing the need for fungicides. Additionally, certain farming practices, such as crop rotation or intercropping, may naturally suppress fungal growth, thereby reducing the need for fungicide applications.

However, our results contrast with survey-based reports from the Central Rift Valley (CRV) of Ethiopia. In the CRV, fungicides are utilized much more intensively due to the region’s focus on horticultural crops like tomatoes and onions, which are highly susceptible to fungal blights (Mengistie et al. 2017). Furthermore, the high reliance on herbicides in the Lake Tana sub-basin, particularly for rice production in the Fogera district, differs from the CRV, where manual weeding by hired laborers is more prevalent in vegetable plots. This underscores how regional crop specializations and specific pest pressures (e.g., rice weeds versus vegetable fungi) dictate the pesticide profile of a watershed.

To synthesize pesticide use patterns with environmental occurrence and ecological relevance, a conceptual table was developed linking active ingredients reported in the Lake Tana sub-basin to their main crop uses, environmental detection locations, and reported ecological risk levels (Table S3). This synthesis integrates survey-based studies documenting pesticide use across different districts with monitoring studies reporting pesticide detections in water, sediment, and fish tissues. Pesticides reported as being used by local farmers but not detected in environmental matrices were not subjected to ecological risk characterization, as the absence of measured environmental concentrations precludes quantitative risk assessment. However, this synthesis also highlights a critical spatial bias in current research. While districts such as Libokemkem, Fogera, and Gondar Zuria in the eastern and northeastern plains have been relatively well-monitored due to their intensive rice and vegetable production, other highly irrigated areas remain significantly underrepresented. Notably, the southern and southwestern parts of the basin, which are home to the Koga irrigation scheme (6,508 ha in Mecha) (Taye et al. 2021) and the Tana Zegie and Tana Wenjeta pump irrigation scheme (Mequanent et al. 2019), lack systematic environmental monitoring. Despite the high agricultural intensity and use of active ingredients in these southern watersheds, the lack of site-specific monitoring data prevents an accurate assessment of the total contaminant load entering the lake from these tributaries. Consequently, there is an urgent need for more intensive, basin-wide monitoring that bridges these geographic gaps to ensure that conservation strategies for Lake Tana’s endemic biodiversity are based on a representative dataset covering all major irrigation entry points.

### Ecological Risk Assessments

#### Legacy Pollutants Versus Current-use Pesticides

The ecological and human health risks identified in the Lake Tana sub-basin arise from two distinct toxicological profiles of legacy organochlorine pesticides (OCPs) and currently applied pesticides. Legacy OCPs, such as DDT, lindane, endosulfan, endrin, and dieldrin, represent a historical burden. Although most were banned decades ago, their high soil organic carbon partition coefficient and lipophilic nature cause them to persist in sediments and bioaccumulate in the fatty tissues of fish (Jayaraj et al. 2016). The risks here are chronic and systemic, driven by the “sink” effect of Lake Tana’s sediment. Current-Use Pesticides (CUPs), chemicals such as imidacloprid (neonicotinoid), oxamyl (carbamate), and flazasulfuron (sulfonylurea) reflect modern agricultural intensification in the Gumara and Ribb irrigation schemes.

### **Risks of Pesticide Body Residues in fish**

**Fig. 3:**
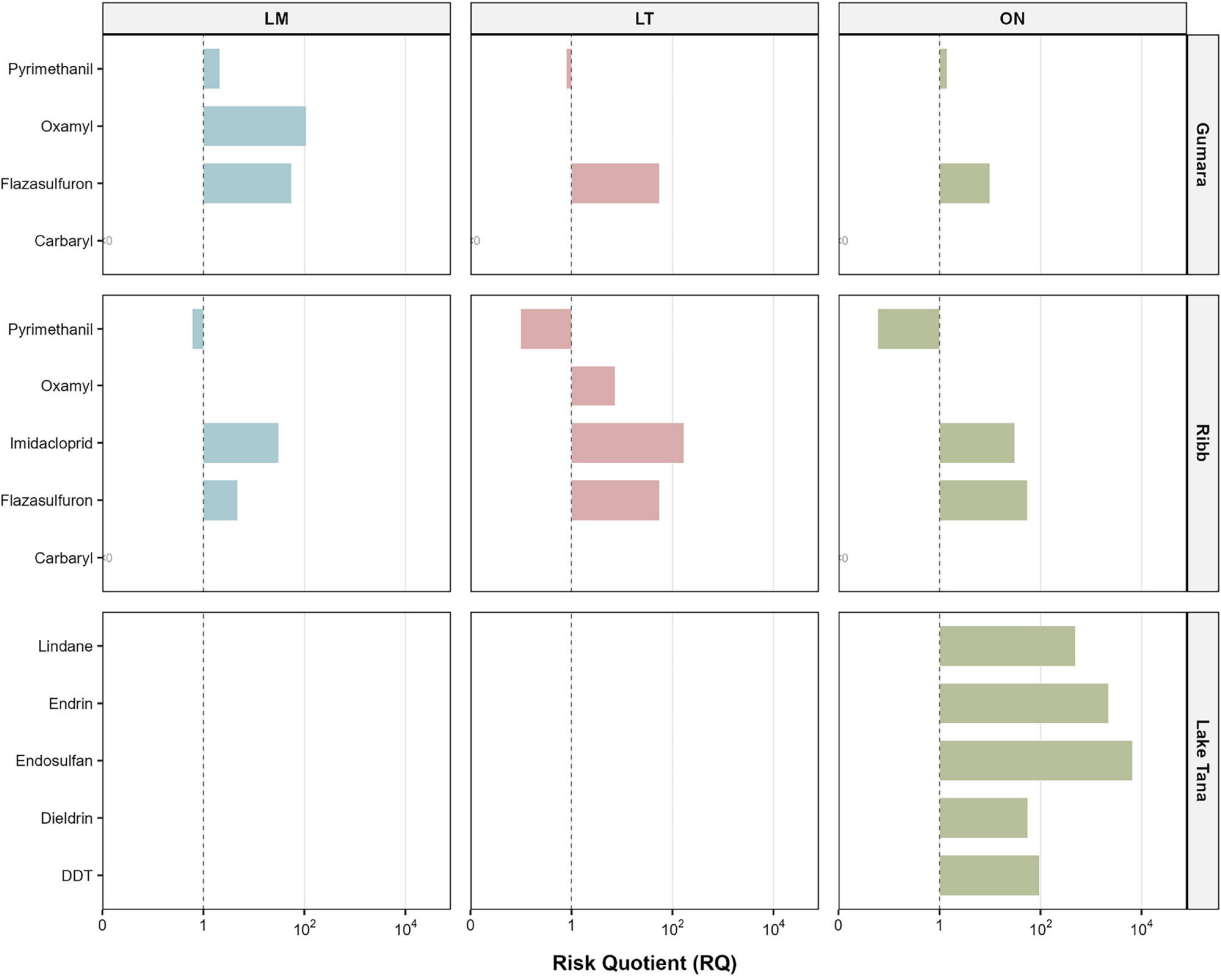
Summary of the RQ calculated based on the body residues of pesticides in fish in the Lake Tana sub-basin. See Table S2 for more detailed information. LM Labeobarbus megastoma, LT Labeobarbus tsanensis, ON Oreochromis niloticus

The environmental risks of pesticide residues to fish species (*Labeobarbus megastoma*, *Labeobarbus tsanensis*, and *Oreochromis niloticus*) in the Gumara River (Fogera district), Ribb River (Fgera abd Libo Kemkem districts) on the eastern side of Lake Tana, and Lake Tana itself were assessed using risk quotients (RQs) derived from measured body burdens (Table S4; Fig. 3). In the Gumara River, oxamyl and flazasulfuron presented the highest risks, posing very high and high risks to *L. megastoma*, respectively (Fig. 3). Flazasulfuron also posed a high risk to *L. tsanensis* and *O. niloticus*. Other pesticides, including pyrimethanil and carbaryl, presented low or no risk (RQ < 1) to the species in this river. In the Ribb River, imidacloprid was the primary concern, posing a high risk to *L. megastoma* and *O. niloticus* and a very high risk to *L. tsanensis* (Fig. 3). Flazasulfuron posed a high risk to *L. tsanensis* and *O. niloticus* and a low risk to *L. megastoma*. The measured concentration of oxamyl posed a low risk to *L. tsanensis*, while pyrimethanil and carbaryl levels posed no risk (RQ < 1) to all species (Fig. 3). In Lake Tana, *O. niloticus* was at risk from measured organochlorines concentrations such as endosulfan, endrin, and lindane posed a very high risk to fish populations, while DDT and dieldrin also posed high risks (Fig. 3).

These results are consistent with broader African studies that emphasize (i) legacy OCPs as ongoing risks for freshwater fish and (ii) currently used pesticides (carbamates, neonicotinoids, sulfonylureas) as emerging concerns. For example, in South African freshwater systems, Ansara-Ross et al. (2012) also reported that OCPs (endosulfan, DDT) remain a priority threat to fish despite bans, while Olisah et al. (2022) showed a strong spatial and seasonal variation of organophosphates in estuarine fish, stressing the role of timing of monitoring and the hydrology of the system under investigation. Similarly, Ogbeide et al. (2015) found that fish from Nigerian rivers accumulated multiple pesticide residues, with carbamates and OCPs frequently exceeding ecological risk thresholds. Together, these studies confirm that the high risks of OCPs to *O. niloticus* in Lake Tana are part of a continent-wide pattern of persistence and bioaccumulation of these legacy pesticides. Although RQs for organochlorine pesticides were explicitly found only for *O. niloticus*, the endemic *Labeobarbus species* of Lake Tana (*L. tsanensis* and *L. megastoma)* may be equally or more vulnerable to these compounds based on their trophic ecology. *L. tsanensis* is predominantly insectivorous while *L. megastoma*, as a largely piscivorous species (Zelalem et al. 2023), occupies a higher trophic position and is therefore expected to experience enhanced exposure through biomagnification. Studies have shown that organochlorine pesticide concentrations increase with trophic level, with insectivorous and piscivorous fish exhibiting higher body burdens than omnivorous species due to dietary transfer and lipid affinity of these compounds (Erdogrul 2005; Eqani et al. 2013; Zhou Shan Shan et al. 2018). Consequently, the high risks observed for *O. niloticus* likely represent a conservative estimate of ecosystem-level risk, and the endemic Labeobarbus species may face comparable or greater long-term threats from legacy OCP contamination in the Lake Tana food web.

The very high risk of oxamyl (a carbamate) to *L. megastoma* as assessed in the Gumara River is similar to a Ghanaian risk assessment, where Onwona-Kwakye et al. (2020) predicted carbamate insecticides as key contributors to aquatic risk using regulatory ecological risk assessment scenarios. Similarly, in their systematic review for sub-Saharan Africa, Fuhrimann et al. (2021) emphasized carbamates among the most frequently applied and risk-relevant pesticide groups. For imidacloprid, the high risks observed for both *L. tsanensis* and *O. niloticus* in the Lake Tana sub-basin are similar to risks assessed using Ethiopia-wide surface water modeling (Teklu et al. 2015; Asefa et al. 2024), which identified neonicotinoids as high-risk drivers for aquatic invertebrates and fish. Risks from flazasulfuron body residues varied by species and river. While high risks from this herbicide were calculated for several fish-sampling site combinations, the background values on which its low PNEC (0.043 µg/kg ww) is based are unknown, as it is likely based on a confidential report supplied by industry to one of the EU regulators (https://www.norman-network.com/nds/ecotox/). Similar results were reported by Tyohemba et al. (2021), who showed that herbicides in South Africa bioaccumulate in fish and potentially disrupt food-web energy flow. Although pyrimethanil body residues posed low or no risk, fungicides have been recognized as sub-lethal stressors in other African ERA studies. For example, Asefa et al. (2024) noted that fungicides pose lower direct risks compared to insecticides but can still impair community dynamics when combined with other pesticide stressors. The low RQs for carbaryl (carbamate) in this study might mask concerns for chronic and mixture effects as observed in Nigerian rivers (Ogbeide et al. 2015). The very high risks to *O. niloticus* posed by endosulfan, lindane, endrin, DDT, and dieldrin body residues are part of a broader African pattern observed with legacy pesticides. Across the continent, OCP residues remain a major ecological threat in South African estuaries, where endosulfan continues to pose risks (Olisah et al. 2022; Ansara-Ross et al. 2012). Their residues also pose risks to fish in Nigerian rivers, where DDT and endosulfan persist at hazardous levels decades after bans (Ogbeide et al. 2015). Legacy OCPs remain ubiquitous drivers of risk in other African aquatic systems as well (Fuhrimann et al. 2021; Asefa et al. 2024), as they bioaccumulate in fish, herewith posing health risks to human consumers, a concern also raised by local farming communities (Orou-Seko et al. 2024).

Unlike most monitoring-based studies performed in Ghana, Nigeria, and South Africa, and the modeling or meta-analyses performed in Ethiopia and across Africa, this assessment was based on measured fish body burdens used to calculate RQs. By capturing organ-specific body residues in muscle and liver tissues, reflecting both bioaccumulation and metabolic processing of pesticide residues. Together, these findings stress the need for African ERA frameworks to integrate both exposure modeling and monitoring and biota-based body-residue approaches to fully characterize risks.

### Human Health Risk Assessment

**Fig. 4:**
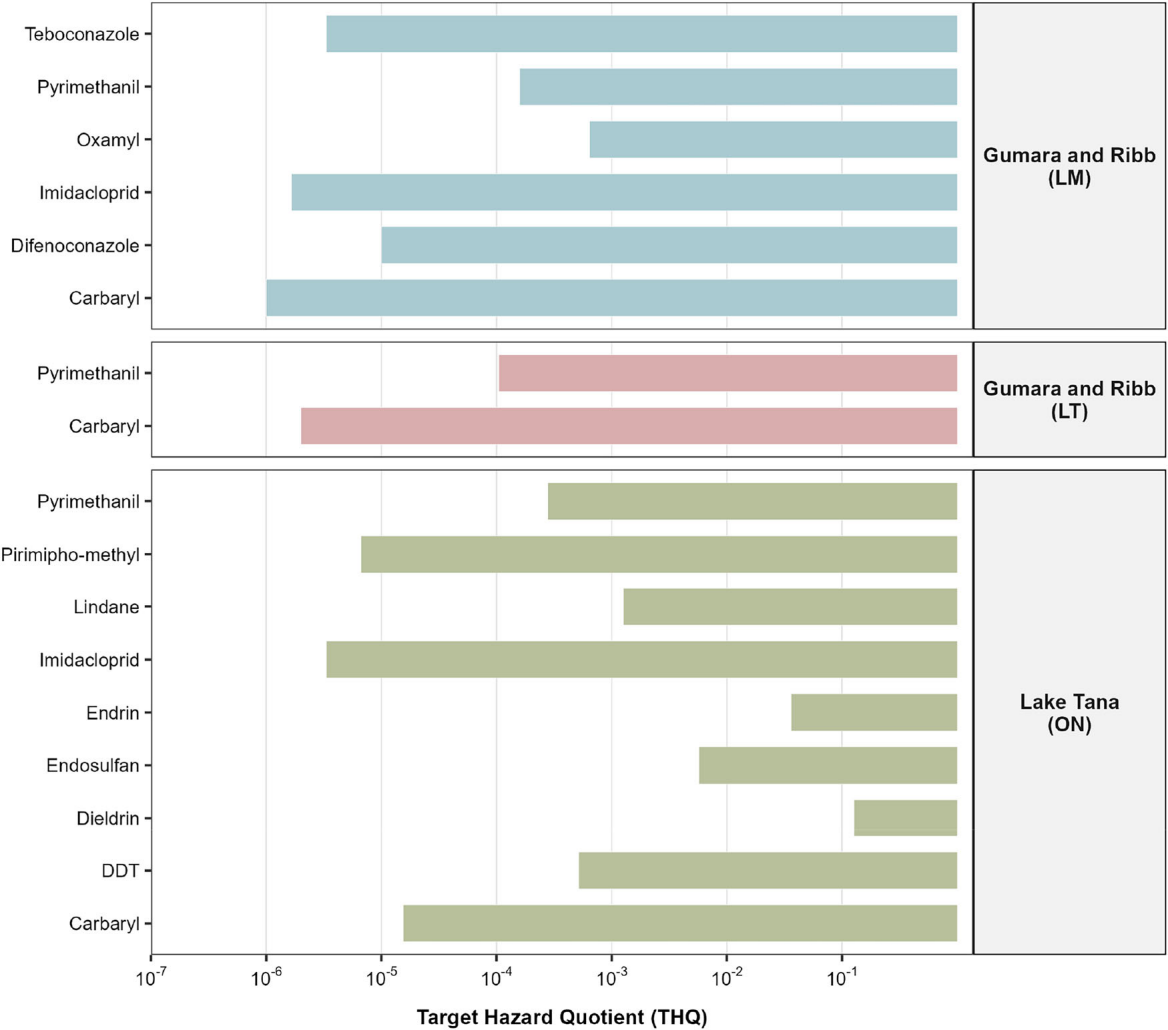
Target Hazard Quotients (THQ) for human health risks associated with pesticide residues in fish muscle from the Gumara and Ribb rivers and Lake Tana. Values are shown for three key species: Labeobarbus megastoma (LM), Labeobarbus tsanensis (LT), and Oreochromis niloticus (ON). While all individual THQ values remain below the safety threshold

THQ values for all detected pesticides are presented in Table S5. At the Gumara and Ribb sites, THQs for current-use pesticides ranged from 0.000001 (carbaryl) to 0.00064 (oxamyl). In Lake Tana, THQs for current-use pesticides ranged from 0.0000033 (imidacloprid) to 0.0002791 (pyrimethanil) (Fig. 4). For legacy organochlorine pesticides in Lake Tana, THQs ranged from 0.000516 (DDT) to 0.1269 (dieldrin). Particularly, dieldrin showed the highest THQ among all pesticides, highlighting its dominant contribution to non-carcinogenic risk (Fig. 4). The cumulative non-carcinogenic risk (Hazard Index, HI) for legacy pesticides was 0.171, while the cumulative HI for current-use pesticides was < 0.001, indicating that overall non-carcinogenic risk remains below the safety threshold (HI < 1).

**Fig. 5:**
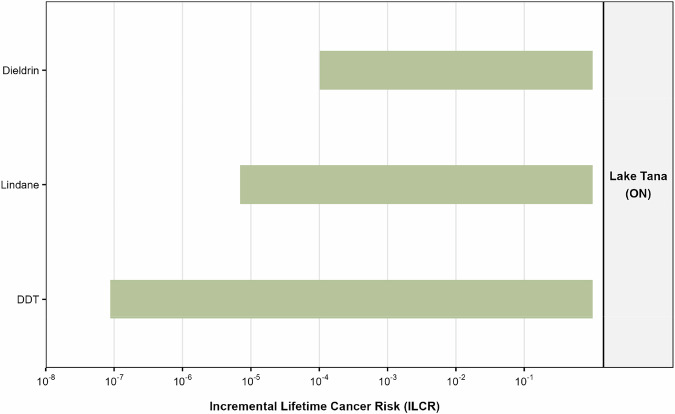
Incremental Lifetime Cancer Risk (ILCR) for Oreochromis niloticus consumers in Lake Tana to legacy organochlorine pesticides

The Incremental Lifetime Cancer Risk (ILCR) was calculated for DDT, lindane, and dieldrin from dietary exposure to *O. niloticus* from Lake Tana. The cumulative ILCR for these three carcinogenic pesticides was 0.000109, slightly above the USEPA action level of 0.0001, indicating potentially unacceptable lifetime cancer risk. Dieldrin is the primary driver of carcinogenic risk, far exceeding the ILCR of other detected pesticides (Fig. 5, Table S5). This study provides a dual-perspective health risk assessment, distinguishing between risks posed by current-use pesticides and those from persistent legacy organochlorines in Lake Tana’s fish. The non-carcinogenic risk assessment showed that all individual THQs were substantially below 1, and the cumulative Hazard Index (HI = 0.171 for legacy pesticides; <0.001 for current-use) confirms that chronic consumption of fish from Lake Tana, Gumara, and Ribb is unlikely to cause systemic health effects. These results are consistent with studies from other Ethiopian water bodies, such as Lake Ziway and northern Rift Valley lakes, where non-carcinogenic risk indices for both organochlorines and current-use pesticides were generally below the threshold of concern, indicating limited immediate systemic risk (Yohannes et al. 2014; Tarekegn et al. 2025). risks, While DDT and lindane posed negligible or tolerable risks, dieldrin contributed the largest carcinogenic risk, slightly exceeding the USEPA ILCR benchmark of 0.0001. The cumulative ILCR (0.000109) indicates that lifetime exposure to carcinogenic residues in O. niloticus may be unacceptable. This pattern is similar to findings from Lake Ziway, where legacy organochlorines such as lindane and DDT and certain OCPs were the primary drivers of cumulative cancer risk in fish (Yohannes et al. 2014; Tarekegn et al. 2025). In contrast, lindane and DDT concentrations reported in the Volta Basin of Ghana were within U.S. EPA regulatory limits, indicating lower associated health risks compared to those observed in the present study (Magna et al. 2022).

### Biodiversity Risk Assessments

The detected pesticides in the water of the Lake Tana sub-basin included the legacy pesticides DDTs, p,p’-DDE, lindane, endosulfan, endrin, dieldrin and O,P’-DDT, as well as cypermethrin and alachlor (Table S6). Bifenthrin and chlorothalonil were reported at levels below the limit of detection. Alachlor was detected only at very low concentrations, resulting in negligible PAF and msPAF values, and was therefore excluded from the risk figures to improve graphical clarity. In contrast, O,P′-DDT could not be included in probabilistic risk calculations due to the absence of an SSD, which prevented PAF estimation.

**Fig. 6:**
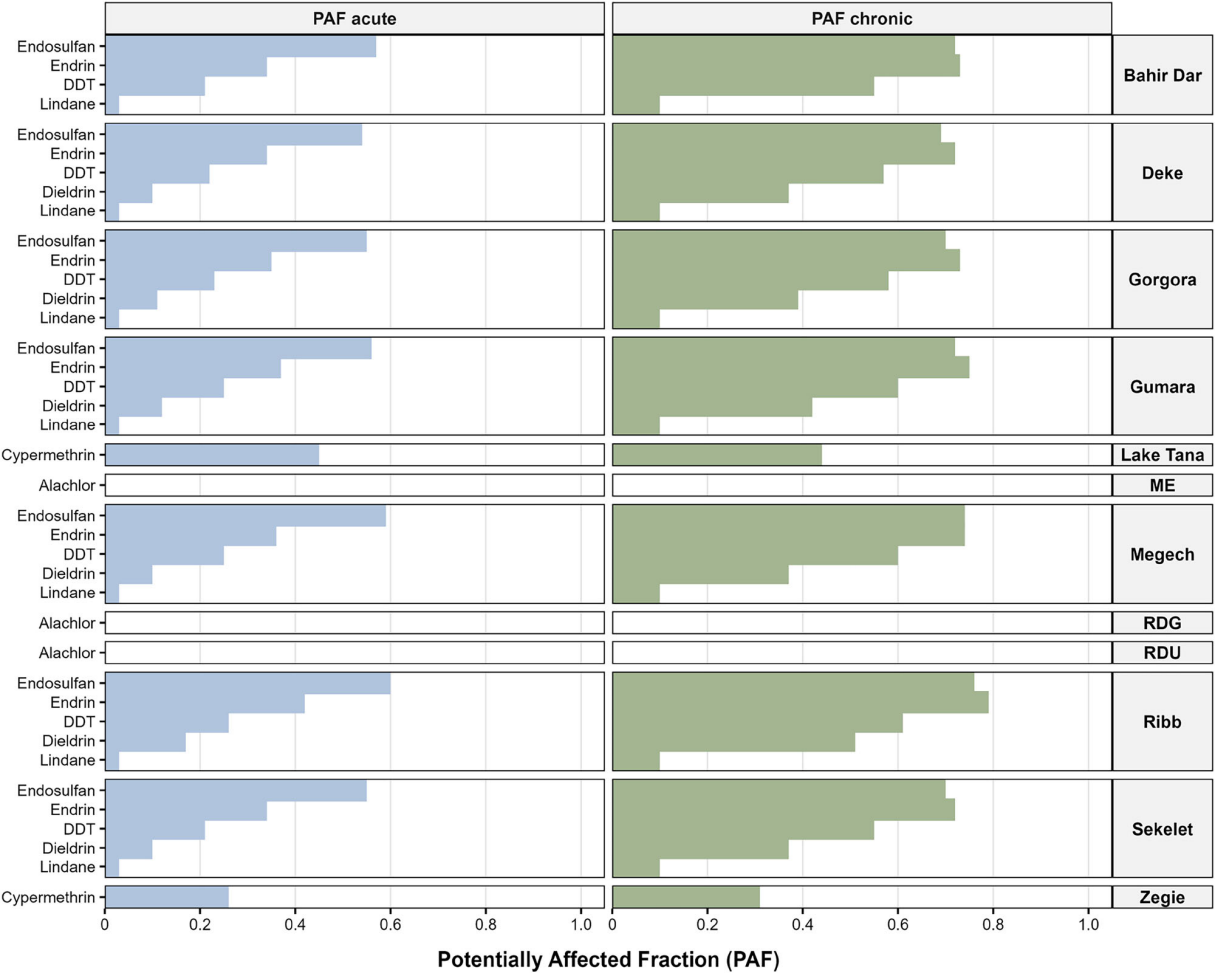
Potentially affected fraction (PAF) values calculated from the measured concentrations of pesticides in the lake, river, and wetland samples (Table S5), arranged from south to north in the lake and upstream to the river mouth in the river, with wetland included as a separate category. The abbreviated sampling sites ME (Megech Wetlands), RDG (Dehena Mesenta Wetlands, sampling point 1), and RDU (Dehena Mesenta Wetlands, sampling point 2) are described in Table S6

The probabilistic risk assessment approach (PAF and msPAF) indicated that endosulfan concentrations posed high ecological risks, particularly in the Ribb River (in Fogera and Libokemkem district) (PAF acute: 0.60, PAF chronic: 0.76), Megech River (in the Dembia district) (PAF acute: 0.59, PAF chronic: 0.74), and Gumara River (Fogera) (PAF acute: 0.56, PAF chronic: 0.72). Within Lake Tana, the highest acute PAF values for endosulfan were observed at Bahir Dar (PAF acute: 0.57, PAF chronic: 0.72) (Fig. 6). Cypermethrin, detected at Zegie (PAF acute: 0.26, PAF chronic: 0.31) and St. George church (LT3) (PAF acute: 0.45, PAF chronic: 0.44), exhibited substantial risks. The highest risks of measured dieldrin (PAF acute: 0.17, PAF chronic: 0.51), endrin (PAF acute: 0.42, PAF chronic: 0.79) and lindane (PAF acute: 0.03, PAF chronic: 0.10) concentrations were all observed for Ribb River, followed by Megech River, Gumara River, Gorgora, Sekelet, Bahir Dar and Deke. p,p’-DDE was frequently detected in both lake and river water bodies, but at lower risk levels (PAF acute: 0.01–0.02, PAF chronic: 0.08–0.10), with the highest concentrations in the northern and southern parts of Lake Tana and at river mouths (Table S6). Wetlands also exhibited traces of p,p’-DDE, indicating pesticide runoff into adjacent aquatic habitats. p,p’-DDE is a breakdown product of DDT and is known for its persistence and bioaccumulation in aquatic ecosystems. Its presence, even at low concentrations, can have long-term effects on aquatic organisms (Giroux et al. 2024).

**Fig. 7:**
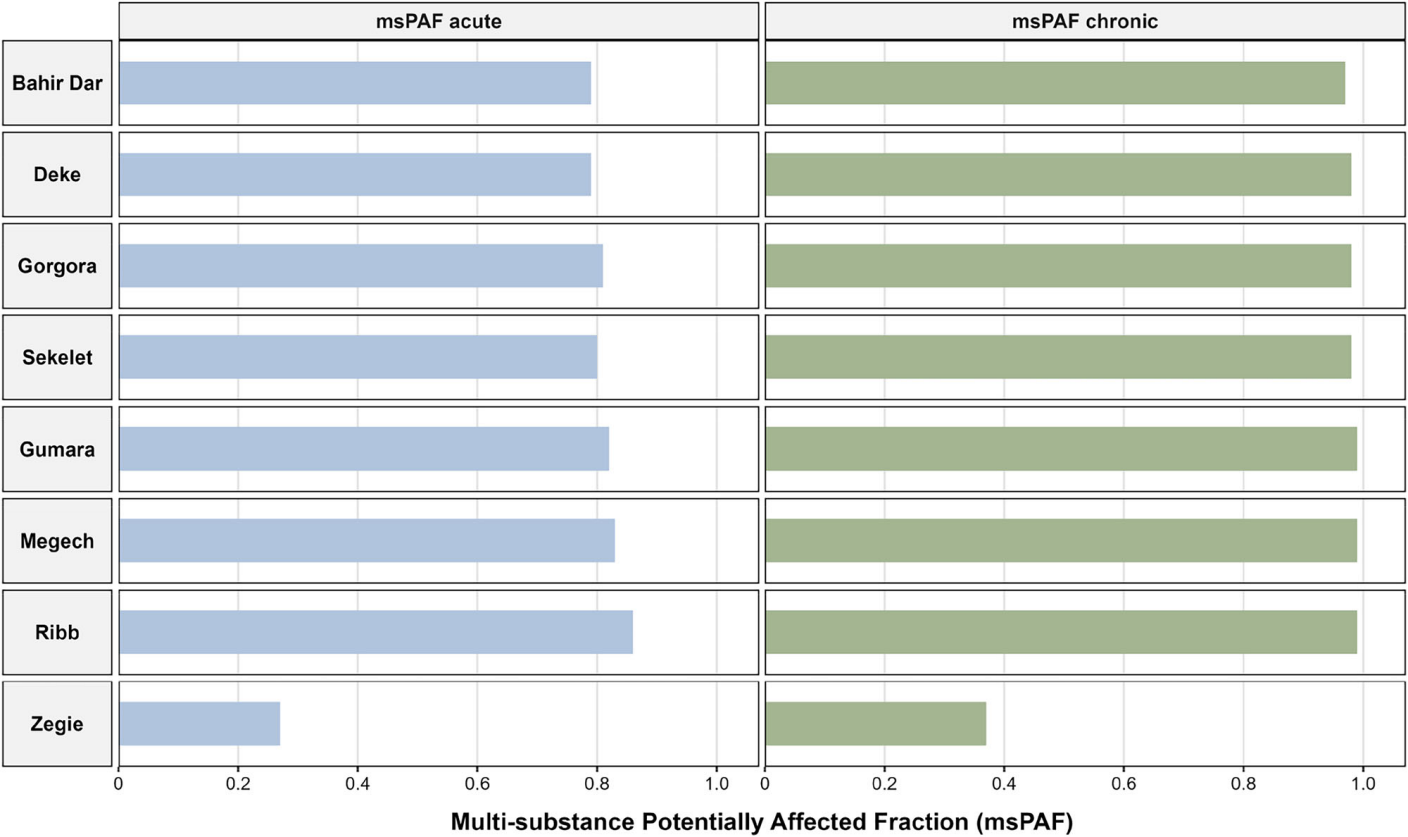
msPAF values in water bodies (lake and river) across various samples. The msPAF acute and chronic values are derived from the combined contributions of pesticides detected in each sample

When multiple substances were combined (msPAF), the ecological risk increased significantly. In the Ribb River, the combined presence of DDTs, dieldrin, endosulfan, endrin, and lindane resulted in high risk levels (msPAF acute: 0.86, msPAF chronic: 0.99), with similar values also calculated for the other sites (Fig. 7). Only for one site in the lake region (Zegie), where only cypermethrin and p,p’-DDE were detected, the combined risk was lower (msPAF acute: 0.27, msPAF chronic: 0.37) (Table S6). The high ecological risks indicated by the PAF and msPAF analyses in the Lake Tana sub-basin are consistent with findings from other African freshwater ecosystems impacted by agricultural pesticide use. For example, in Lake Ziway, Ethiopia, Merga et al. (2021) reported moderate to high ecological risks from current-use pesticides using a probabilistic risk assessment approach, although legacy organochlorine pesticides were not included in that study. The substantially higher msPAF values observed in the present study highlight the additional contribution of co-occurring legacy OCPs to mixture toxicity in the Lake Tana system. Importantly, there is very limited research in Ethiopia and across African freshwater systems that applies mixture-based probabilistic risk assessment approaches such as PAF and msPAF, which limits regional comparisons. These findings also align with the growing consensus that mixture toxicity is a dominant driver of pesticide impacts in aquatic ecosystems. Dietrich et al. (2022) demonstrated that regulatory risk assessments based solely on single-substance evaluations frequently underestimate ecological risks and that pragmatic mixture-based approaches, grounded in concentration addition and Species Sensitivity Distributions (SSDs), provide a more realistic assessment of impacts on aquatic communities.

### Risks of Sediment-bound Pesticides

**Fig. 8:**
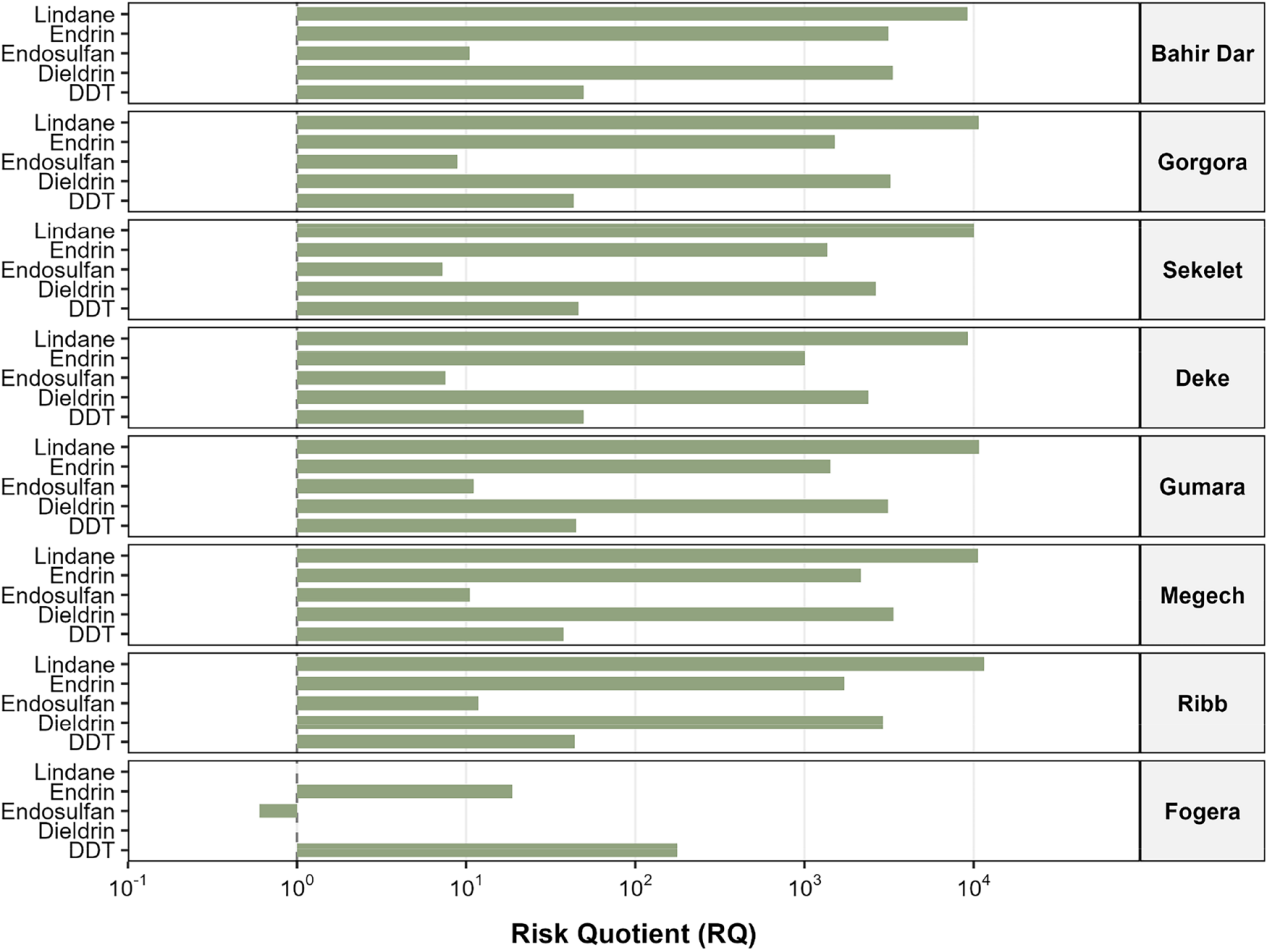
Sediment-associated ecological risk quotients (RQs) for legacy organochlorine pesticides across eight sampling sites in the Lake Tana sub-basin

The calculated risk quotients showed ecological risks associated with sediment-bound pesticides across all sampling sites in the Lake Tana sub-basin (Fig. 8). Among the assessed pesticides, lindane consistently exhibited the highest RQ values, exceeding 9000 at all sites, with maximum values observed at Ribb and Gumara (Fig. 8, Table S7). Similarly, endrin and dieldrin showed very high RQ values, ranging from 1000 to 3300. In contrast, DDT and endosulfan displayed moderate to high risks, with RQ values generally exceeding unity across sites. Although the Fogera site showed comparatively lower RQ values, DDT still exceeded risk thresholds.

The high RQ values for organochlorine pesticides (OCPs) in sediments reflect the persistent, hydrophobic, and bioaccumulative nature of these pesticides, which promotes their long-term retention in sediments. Similar patterns have been reported in other Ethiopian freshwater systems, including the Awash River Basin (Dirbaba et al. 2018) and the Tekeze Dam (Teklit, 2016), Lake Ziway (Merga et al. 2021), Akaki River (Kassegne et al. 2020), and Lake Awassa (Yohannes et al. 2013; Wm-Bekele et al. 2024), which point out the widespread legacy of historical pesticides used. Notably, the RQ values of pesticides are markedly higher than those reported for other Ethiopian water bodies. For example, sediment concentrations in the Awash River were reported at 8.33 µg kg⁻¹ for lindane, 13.98 µg kg⁻¹ for DDT, 0.81 µg kg⁻¹ for endosulfan, and 1.0 µg kg⁻¹ for endrin (Dirbaba et al. 2018). Similarly, in Lake Ziway, where endosulfan levels reached 2.69 µg kg⁻¹ (Merga et al. 2021), the calculated risks remain substantially lower than those observed in the Lake Tana sub-basin. The high RQs for lindane, endrin, and dieldrin found in this study indicate a significant potential for adverse effects on benthic invertebrates, such as oligochaetes, chironomids, and amphipods. These organisms play critical roles in sediment food webs and nutrient cycling; consequently, their impairment could trigger cascading effects in the ecosystem (Zhao et al. 2009; Jayaraj et al. 2016).

Beyond Ethiopia, studies across Africa show comparable risks from sediment-associated pesticides in freshwater ecosystems. For instance, Darko et al. (2008) reported RQ values indicating high risk for lindane, moderate risk for DDT, and low risk for dieldrin and endosulfan in Ghanaian lake sediments. Similarly, the application of threshold-based sediment quality benchmarks identified dieldrin as a major ecological threat in the Ugandan portion of Lake Victoria (Wasswa et al. 2011). Furthermore, Buah-Kwofie and Humphries (2017) reported that OCP concentrations in sediments from the iSimangaliso Wetland Park, South Africa, were among the highest recorded both nationally and globally in recent decades. The authors emphasized that sediments within lakes and wetlands act as substantial reservoirs for contaminants, posing significant ecotoxicological threats to internationally recognized biodiversity hotspots. They underscored the urgent need for detailed bioaccumulation and toxicological assessments in such regions. These external findings are highly consistent with the present study, which identifies lindane, endrin, and dieldrin as the primary compounds of concern in the Lake Tana sub-basin.

### Pesticide Misuse and Overuse Practices

The Lake Tana sub-basin is highly productive, supporting staple and cash crops such as teff, millet, maize, and vegetables (Merkuz 2017; Agmas and Adugna 2020; Taye et al. 2021). Intensive pesticide use, with sometimes up to twenty-six applications per season, is common, particularly on irrigated cash crops (Merkuz 2017; Agmas and Adugna, 2020; Abaineh et al. 2024). Despite national Integrated Pest Management (IPM) guidelines and awareness campaigns (FDRE MOA & RDCPD 2006), adoption remains limited.

Farmers in the sub-basin often lack formal education and training on pesticide safety, leading to misuse and risky application practices (Agmas and Adugna 2020; Asmare et al. 2022). Economic constraints further drive reliance on older, cheaper, and more toxic pesticides (Hu 2020). Improper application contributes significantly to environmental contamination in the region (Merkuz 2017; Agmas and Adugna 2020; Abaineh et al. 2024). Climate change is also increasing pest pressure, further incentivizing pesticide use (IPCC 2001; Palikhe 2007).

### Management Implications for Legacy and Current-use Pesticides

To reduce risks from current-use pesticides, strategies should focus on education, monitoring, and promoting safer practices:

- Strengthen farmer training and extension services, including farmer field schools and peer-to-peer learning (Asmare et al. 2022).
- Promote Integrated Pest Management (IPM) tailored to local conditions, including crop rotation, diversification, and the use of botanical pesticides (Liebman and Dyck, 1993; Isman, 2006; Reganold and Wachter, 2016).
- Introduce technological solutions such as GPS-guided sprayers and drones, buffer zones near water bodies, and precision application to reduce environmental contamination (Zhang et al. 2002; Arora et al. 2010; Matthews 2006).
- Enhance pest monitoring and forecasting systems to reduce unnecessary pesticide applications (Stehle and Schulz 2015).

For legacy pesticides, the following strategies could be applied:

- Management strategies for legacy organochlorine pesticides (OCPs) differ from current-use pesticides due to their persistence, bioaccumulation, and environmental mobility;
- Identify and monitor hotspots of legacy contamination in rivers, wetlands, and sediments (Tomei et al. 2025).
- Strengthen regulatory enforcement and proper disposal of banned pesticides to prevent further contamination (Alemayehu 2012; Mengistie 2016).
- Promote remediation and containment strategies, such as vegetative buffer zones, sediment dredging where feasible, and limiting agricultural runoff near sensitive water bodies (Pimentel 2005; Arora et al. 2010).
- Conduct public awareness campaigns targeting farmers and local communities on the long-term risks of legacy pesticides and the importance of avoiding illegal use.

## Conclusions

The findings highlight significant environmental risks from pesticide use as assessed through residues in fish, water, and sediment within the Lake Tana sub-basin. These findings underscore the urgent need for integrated management strategies that distinguish between legacy and current-use pesticides. Reducing risks requires stricter regulation and enforcement, promoting safer alternatives and IPM, farmer education, and mitigating underlying drivers such as economic constraints, lack of awareness, and climate-related pest pressures. Sediment remediation and long-term monitoring are critical for legacy pesticide hotspots.

## Supplementary information

Supplementary information

## Data Availability

The data analyzed in this study are provided in the Supplementary Information file.
