# Supplementary material for: Environmental Risks of Pesticide Residues in the Lake Tana Sub-basin, Ethiopia: A Review
Source: Environ Manage. 2026 Feb 18;76(3):108. doi: 10.1007/s00267-026-02399-z (PMC12916960; doi:10.1007/s00267-026-02399-z)
Supplement: Supplementary file 1 — Supplementary information [file 267_2026_2399_MOESM1_ESM.docx]

**Environmental risks of pesticide residues in the Lake Tana sub-basin, Ethiopia: a review**

Banchiamlak Getnet Admasu ^1,2^, Kaisheng Yao ^1^, Goraw Goshu Yemer ^2,3^, Paul J. Van den Brink ^1,*^

^1^ Aquatic Ecology and Water Quality Management Group, Wageningen University, P.O. Box 47, 6700AA Wageningen, the Netherlands

^2^ College of Agriculture and Environmental Sciences, Bahir Dar University, P.O. Box 79, Bahir Dar, Ethiopia

^3^ Blue Nile Water Institute, Bahir Dar University, P. O. Box 79, Bahir Dar, Ethiopia

* Corresponding author. Wageningen University, P.O. Box 47, 6700, AA Wageningen, the Netherlands.

E-mail address: [paul.vandenbrink@wur.nl](mailto:paul.vandenbrink@wur.nl); ORCID: 0000-0002-7241-4347


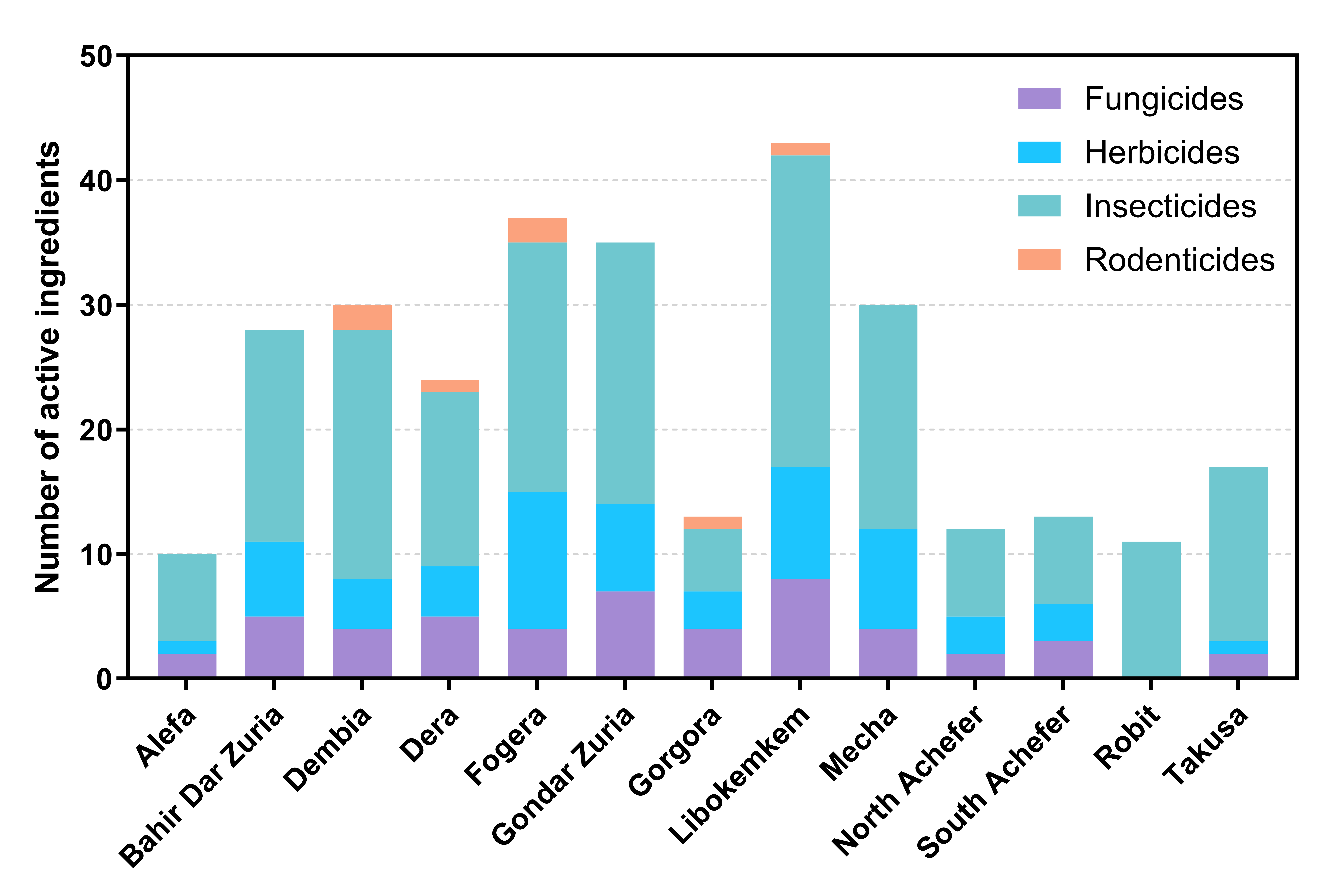


**Fig. S1.** Reported pesticides in 13 districts in the Lake Tana sub-basin, based on the kind of pest they regulate. Source: (Begna, 2015; Agmas and Adugna, 2020; Asmare et al., 2022; Sishu et al., 2022; Abaineh et al., 2023; Alebachew et al., 2023; Tassew et al., 2023).

**Table S1.** Summary of irrigation schemes in the Lake Tana sub-basin.

| Irrigation scheme name | Irrigated land (ha) | Command area (ha) | Operational status | Location (Sub-basin / area) | References |
| --- | --- | --- | --- | --- | --- |
| Megech-Seraba | 566 | 4995 | Partially operational) | North (Gondar) | ADSWE, 2017; Taye et al., 2021 |
| Tana Asrate | No data | 1333 | Local small‑scale operation | Northwest | ADSWE, 2017; Mequanent et al., 2019 |
| Tana Mekonta | No data | 1140 | Local small‑scale operation | Northwest | ADSWE, 2017; Mequanent et al., 2019 |
| Tana Wenjeta | 48.5 | 4849 | Local small‑scale operation | South | ADSWE, 2017; Mequanent et al., 2019; Taye et al., 2021; |
| Tana Zegie | No data | 1265 | Local small‑scale operation | South (Zegie Peninsula area) | ADSWE, 2017 |
| Shina | 100 | 100 | Variable (Small-scale pump/diversion) | East (Fogera Floodplain) | Abera et al., 2019 |
| Bebekis | 61 | 60 | Variable (Small-scale pump/diversion) | East (Fogera Floodplain) | Abera et al., 2019 |
| Kuhar-Michael | No data | No data | No data | East (Fogera Floodplain) | Eguavoen et al., 2012 |
| Koga | 6,508 | 7000 | Fully operational | Southwest (Mecha Woreda) | ADSWE, 2017; Taye et al., 2021 |

**Table S2.** Types of pesticides used in 13 districts of the Lake Tana sub-basin, by target pest.

| Sampling sites | Types of active ingredients | Chemical classes | References |
| --- | --- | --- | --- |
| Gorgora | Endosulfan | Insecticides | Agmas and Adugna, 2020 |
|  | Diazinon |  |  |
|  | Dimethoate |  |  |
|  | Malathion |  |  |
|  | Deltamethrin |  |  |
| Dembia | Endosulfan | Insecticides | Abera et al., 2022 |
|  | Diazinon |  |  |
|  | Dimethoate |  |  |
|  | Malathion |  |  |
|  | Deltamethrin |  |  |
|  | Chlorpyrifos |  |  |
|  | Lambda cyhalothrin |  |  |
|  | Profenofos |  |  |
|  | Bifenthrin |  |  |
|  | Permethrin |  |  |
|  | Dicofol |  |  |
|  | Temephos |  |  |
|  | Carbaryl |  |  |
|  | Aluminium phosphide |  |  |
|  | Indoxacarb |  |  |
|  | Abamectin |  |  |
|  | Tebufenozide |  |  |
|  | Terbufos |  |  |
|  | Methiocarb |  |  |
| Fogera | Endosulfan | Insecticides | Agmas and Adugna, 2020; Abera et al., 2022; Alebachew et al., 2023; Tassew et al., 2023; Abaineh et al., 2024 |
|  | Diazinon |  |  |
|  | Dimethoate |  |  |
|  | Malathion |  |  |
|  | Deltamethrin |  |  |
|  | Chlorpyrifos |  |  |
|  | Lambda cyhalothrin |  |  |
|  | Profenofos |  |  |
|  | Imidacloprid |  |  |
|  | Azadirachtin |  |  |
|  | Acetamiprid |  |  |
|  | Pirimiphos-methyl |  |  |
|  | Phenthoate |  |  |
|  | Fenthion |  |  |

| Sampling sites | Types of active ingredients | Chemical classes | References |
| --- | --- | --- | --- |
| Libokemkem | Diazinon | Insecticides | Agmas and Adugna, 2020; Abera et al., 2022; Tassew et al., 2023 |
|  | Methomyl |  |  |
|  | Endosulfan |  |  |
|  | Dimethoate |  |  |
|  | Malathion |  |  |
|  | Deltamethrin |  |  |
|  | Chlorpyrifos |  |  |
|  | Lambda cyhalothrin |  |  |
|  | Profenofos |  |  |
|  | Bifenthrin |  |  |
|  | Thiamethoxam |  |  |
|  | Permethrin |  |  |
|  | Dicofol |  |  |
|  | Carbaryl |  |  |
|  | Indoxacarb |  |  |
|  | Flubendiamide |  |  |
|  | Spiromesifen |  |  |
|  | Novaluron |  |  |
|  | Fenoxycarb |  |  |
|  | Imidacloprid |  |  |
|  | Azadirachtin |  |  |
|  | Acetamiprid |  |  |
|  | Pirimiphos-methyl |  |  |
|  | Phenthoate |  |  |
|  | Fenthion |  |  |
| Dera | Diazinon | Insecticides | Agmas and Adugna, 2020; Abera et al., 2022; Asmare et al., 2022 |
|  | Endosulfan |  |  |
|  | Dimethoate |  |  |
|  | Malathion |  |  |
|  | Deltamethrin |  |  |
|  | Chlorpyrifos |  |  |
|  | Lambda cyhalothrin |  |  |
|  | Profenofos |  |  |
|  | Bifenthrin |  |  |
|  | Thiamethoxam |  |  |
|  | Temephos |  |  |
|  | Clothianidin |  |  |
|  | Methiocarb |  |  |
| Bahir Dar Zuria | Diazinon | Insecticides | Abera et al. 2022; Tassew et al., 2023 |
|  | Chlorpyrifos |  |  |
|  | Dimethoate |  |  |
|  | Lambda cyhalothrin |  |  |
|  | Profenofos |  |  |
|  | Bifenthrin |  |  |
|  | Malathion |  |  |
|  | Imidacloprid |  |  |
|  | Azadirachtin |  |  |
|  | Acetamiprid |  |  |
|  | Deltamethrin |  |  |
|  | Endosulfan |  |  |
|  | Pirimiphos-methyl |  |  |
|  | Heptachlor |  |  |
|  | Phenthoate |  |  |
|  | Fenthion |  |  |
| Sampling sites | Types of active ingredients | Chemical classes | References |
| North Achefer | Chlorpyrifos | Insecticides | Abera et al., 2022 |
|  | Malathion |  |  |
|  | Dimethoate |  |  |
|  | Lambda cyhalothrin |  |  |
|  | Deltamethrin |  |  |
|  | Profenofos |  |  |
|  | Bifenthrin |  |  |
| South Achefer | Diazinon | Insecticides | Abera et al., 2022 |
|  | Chlorpyrifos |  |  |
|  | Malathion |  |  |
|  | Dimethoate |  |  |
|  | Lambda cyhalothrin |  |  |
|  | Deltamethrin |  |  |
|  | Bifenthrin |  |  |
| Mecha | Diazinon | Insecticides | Begna, 2015; Abera et al., 2022; Tassew et al., 2023 |
|  | Profenofos |  |  |
|  | Malathion |  |  |
|  | Imidacloprid |  |  |
|  | Azadirachtin |  |  |
|  | Acetamiprid |  |  |
|  | Lambda cyhalothrin |  |  |
|  | Deltamethrin |  |  |
|  | Endosulfan |  |  |
|  | Chlorpyrifos |  |  |
|  | Pirimiphos-methyl |  |  |
|  | Dimethoate |  |  |
|  | Phenthoate |  |  |
|  | Fenthion |  |  |
|  | Heptachlor |  |  |
|  | Bifenthrin |  |  |
| Alefa | Diazinon | Insecticides | Abera et al., 2022 |
|  | Chlorpyrifos |  |  |
|  | Malathion |  |  |
|  | Dimethoate |  |  |
|  | Deltamethrin |  |  |
|  | Profenofos |  |  |
|  | Bifenthrin |  |  |
|  | Methomyl |  |  |
| Takusa | Diazinon | Insecticides | Abera et al., 2022 |
|  | Chlorpyrifos |  |  |
|  | Malathion |  |  |
|  | Dimethoate |  |  |
|  | Lambda cyhalothrin |  |  |
|  | Deltamethrin |  |  |
|  | Profenofos |  |  |
|  | Bifenthrin |  |  |
|  | Thiamethoxam |  |  |
|  | Permethrin |  |  |
|  | Isofenphos |  |  |
|  | Flubendiamide |  |  |
|  | Novaluron |  |  |
|  | Tefluthrin |  |  |
| Gondar Zuria | Diazinon | Insecticides | Abera et al., 2022; Tassew et al., 2023 |
|  | Malathion |  |  |
|  | Lambda cyhalothrin |  |  |
|  | Deltamethrin |  |  |
|  | Profenofos |  |  |
|  | Bifenthrin |  |  |
|  | Isofenphos |  |  |
|  | Clothianidin |  |  |
|  | Tebufenozide |  |  |
|  | Tefluthrin |  |  |
|  | Imidacloprid |  |  |
|  | Azadirachtin |  |  |
|  | Acetamiprid |  |  |
|  | Endosulfan |  |  |
|  | Chlorpyrifos |  |  |
|  | Pirimiphos-methyl |  |  |
|  | Dimethoate |  |  |
|  | Phenthoate |  |  |
|  | Fenthion |  |  |
| Robit | Chlorpyrifos | Insecticides | Sishu et al., 2022 |
|  | Profenofos |  |  |
|  | Dimethoate |  |  |
|  | Endosulfan |  |  |
|  | Diazinon |  |  |
|  | DDT |  |  |
|  | Malathion |  |  |
|  | Imidacloprid |  |  |
|  | Lambda cyhalothrin |  |  |
|  | Propiconazole |  |  |
|  | Mancozeb |  |  |
| Fogera | Tebuconazole | Fungicides | Abera et al., 2022; Abaineh et al., 2024 |
|  | Metalaxyl |  |  |
|  | Mancozeb |  |  |
| Libokemkem | Propiconazole | Fungicides | Agmas and Adugna, 2020; Abera et al., 2022; Tassew et al., 2023 |
|  | Triadimefon |  |  |
|  | Mancozeb |  |  |
|  | Chlorothalonil |  |  |
| Dera | Chlorothalonil | Fungicides | Agmas and Adugna, 2020; Abera et al., 2022; Tassew et al., 2023 |
|  | Mancozeb |  |  |
|  | Propiconazole |  |  |
| Bahir Dar Zuria | Mancozeb | Fungicides | Abera et al., 2022; Tassew et al., 2023 |
|  | Chlorothalonil |  |  |
|  | Propiconazole |  |  |
|  | Triadimefon |  |  |
|  | Metalaxyl |  |  |
| North Achefer | Mancozeb | Fungicides | Abera et al., 2022 |
|  | Metalaxyl |  |  |
| South Achefer | Mancozeb | Fungicides | Abera et al., 2022 |
|  | Metalaxyl |  |  |
|  | Chlorothalonil |  |  |
| Mecha | Chlorothalonil | Fungicides | Abera et al., 2022; Tassew et al., 2023 |
|  | Mancozeb |  |  |
|  | Propiconazole |  |  |
|  | Triadimefon |  |  |
| Alefa | Chlorothalonil | Fungicides | Abera et al., 2022 |
|  | Mancozeb |  |  |
| Takusa | Mancozeb | Fungicides | Abera et al., 2022 |
|  | Chlorothalonil |  |  |
| Gondar Zuria | Triticonazole | Fungicides | Abera et al., 2022; Tassew et al., 2023 |
|  | Propiconazole |  |  |
|  | Chlorothalonil |  |  |
|  | Mancozeb |  |  |
|  | Metalaxyl |  |  |
|  | Triadimefon |  |  |
| Gorgora | 2,4-D amine | Herbicides | Agmas and Adugna, 2020 |
| Dembia | 2,4-D amine | Herbicides | Abera et al., 2022 |
|  | Pyroxsulam |  |  |
|  | 2,4-D |  |  |
| Fogera | 2,4-D amine | Herbicides | Agmas and Adugna, 2020; Abera et al., 2022; Alebachew et al., 2023; Tassew et al., 2023; Abaineh et al., 2024 |
|  | Atrazine |  |  |
|  | 2,4-D |  |  |
|  | Alachlor |  |  |
|  | Glyphosate |  |  |
|  | Glyphosate-isopropylammonium |  |  |
|  | Pyroxsulam |  |  |
|  | Carfentrazone-ethyl |  |  |
|  | Butachlor |  |  |
| Libokemkem | 2,4-D amine | Herbicides | Agmas and Adugna, 2020; Abera et al., 2022; Tassew et al., 2023 |
|  | 2,4-D |  |  |
|  | Atrazine |  |  |
|  | Glyphosate |  |  |
|  | Glyphosate-isopropylammonium |  |  |
|  | Pyroxsulam |  |  |
| Dera | 2,4-D amine | Herbicides | Agmas and Adugna,2020; Abera et al., 2022 |
|  | 2,4-D |  |  |
| Bahir Dar Zuria | Glyphosate | Herbicides | Abera et al., 2022; Tassew et al., 2023 |
|  | Atrazine |  |  |
|  | 2,4-D |  |  |
|  | Glyphosate-isopropylammonium |  |  |
|  | Pyroxsulam |  |  |
|  | Butachlor |  |  |
|  | Atrazine |  |  |
| North Achefer | 2,4-D | Herbicides | Abera et al., 2022 |
|  | Glyphosate |  |  |
|  | Atrazine |  |  |
| South Achefer | Atrazine | Herbicides | Abera et al., 2022 |
|  | 2,4-D |  |  |
|  | Glyphosate |  |  |
| Mecha | Atrazine | Herbicides | Abera et al., 2022; Tassew et al., 2023 |
|  | Butachlor |  |  |
|  | 2,4-D amine |  |  |
|  | 2,4-D |  |  |
|  | Glyphosate |  |  |
|  | Glyphosate-isopropylammonium |  |  |
|  | Pyroxsulam |  |  |
| Alefa | 2,4-D | Herbicides | Abera et al., 2022 |
| Takusa | 2,4-D | Herbicides | Abera et al., 2022 |
| Gondar Zuria | 2,4-D | Herbicides | Abera et al., 2022; Tassew et al., 2023 |
|  | Atrazine |  |  |
|  | Butachlor |  |  |
|  | Glyphosate |  |  |
|  | Glyphosate-isopropylammonium |  |  |
|  | Pyroxsulam |  |  |
| Gorgora | Zinc Phosphide | Rodenticides | Agmas and Adugna, 2020 |
| Dembia | Aluminium phosphide | Rodenticides | Abera et al., 2022 |
|  | Zinc Phosphide |  |  |
| Fogera | Zinc Phosphide | Rodenticides | Abaineh et al., 2024 |
|  | Aluminium phosphide |  |  |
| Libokemkem | Zinc Phosphide | Rodenticides | Agmas and Adugna, 2020 |
| Dera | Zinc Phosphide | Rodenticides | Agmas and Adugna, 2020 |

Reported pesticides in 13 districts of the Lake Tana sub-basin based on the kind of pest they regulate. Source: Begna (2015), Agmas and Adugna (2020), Agmas and Adugna (2022), Abera et al. (2022), Tibebe et al. (2022), Sishu et al. (2022), Tassew et al. (2023), Alebachew et al. (2023), Abaineh et al. (2023) and Abaineh et al. (2024).

**Table S3**. Conceptual framework connecting pesticide active ingredients, crop types, detection matrices, source, and ecological risk

| Pesticide | Chemical classes | Main crop use (survey-based) | Environmental matrix | Ecological risk level | District/source of pesticides | References |
| --- | --- | --- | --- | --- | --- | --- |
| Endosulfan | Insecticides | Onion, Tomato, Pepper, Maize,  Chickpea, Grass pea, Wheat, Teff and Khat | Fish, water and Sediment | Very high | Fogera, Libokemkem and Gorgora | Agmas and Adugna, 2020 |
| Aluminium phosphide |  | Chickpea | Survey | N/A | Fogera, Libokemkem, Gorgora | Agmas and Adugna, 2020; Abaineh et al., 2024 |
| DDT |  | Historically used | Fish, water and Sediment | Very high | Fogera, Libokemkem Dembia and Dera | Tibebe et al, 2022 |
| Chlorpyrifos |  | Onion, Tomato, Pepper, Maize,  Chickpea, Grass pea, Wheat, Teff and Khat | Survey | N/A | Fogera, Libokemkem, Gorgora,  Bahir Dar  Zuria, North  Achefer, South  Achefer,  Mecha, Alefa, Takusa, Dembia, Dera, Gonder Zuria | Agmas and Adugna, 2020; Abera et al., 2022 |
| Diazinon |  | Onion, Tomato, Pepper, Maize,  Chickpea, Grass pea, Wheat, Teff and Khat | Survey | N/A | Fogera, Dera, Libokemkem and Gorgora | Agmas and Adugna, 2020 |
| Dimethoate |  | Onion, Tomato, Pepper, Maize,  Chickpea, Grass pea, Wheat, Teff and Khat | Survey | N/A | Fogera, Dera, Libokemkem and Gorgora | Agmas and Adugna, 2020; Abaineh et al., 2024 |
| Fenitrothion |  | Vetch, Cabbage and Maize | Survey | N/A | Fogera | Abaineh et al., 2024 |
| Fenthion |  | Sorghum, Maize | Survey | N/A | Mecha, Bahir Dar Zuria, Fogera, Libokemkem and  Gondar Zuria | Tassew et al., 2023 |
| Isofenphos |  | N/A | Survey | N/A | Gondar zuria and Takusa | Abera et al., 2022 |
| Malathion |  | Vetch, Cabbage, Maize, Chickpea, Pepper and Onion | Survey | N/A | Fogera,Libokemkem, Dera and Gorgora, | Agmas and Adugna, 2020; Abaineh et al., 2024 |
| Pirimiphos-methyl |  | Sorghum, Maize, Green mung and  Grass pea | Fish | No risk | Mecha, Bahir Dar Zuria, Fogera, Libokemkem and  Gondar Zuria | Tassew et al., 2023 |
| Profenofos |  | Vetch, Rice, Potato ,Garlic, Tomato, Cabbage and Onion | Survey | N/A | Mecha, Bahir Dar Zuria, Fogera, Libokemkem and  Gondar Zuria | Abaineh et al., 2024 |
| Temephos |  | N/A | Survey | N/A | Takusa and Dembia | Abera et al., 2022 |
| Terbufos |  | N/A | Survey | N/A | Gondar zuria and Dembia | Abera et al., 2022 |
| Carbaryl |  | N/A | Fish | Low risk | Takusa , Gondar zuria | Abera et al., 2022 |
| Methiocarb |  | N/A | Survey | N/A | Dera and Dembia | Abera et al., 2022 |
| Bifenthrin |  | N/A | Water | No risk | Bahir  Dar  Zuria,  North  Achefer,  South  Achefer,  Mecha, Alefa Takusa,  Dembia,  Dera ,Libokemkem  and Gondar  Zuria | Abera et al., 2022 |
| Deltamethrin |  | Onion, Tomato, Pepper, Maize,  Chickpea, Grass pea, Wheat, Teff and Khat | Survey | N/A | Fogera, Dera, Libokemkem and Gorgora | Agmas and Adugna, 2020 |
| Lambda cyhalothrin |  | Vetch, Rice, Potato and Garlic | Survey | N/A | Fogera | Abaineh et al., 2024 |
| Dicofol |  | N/A | Survey | N/A | Takusa,Dembia, Libokemkem | Abera et al., 2022 |
| Permethrin |  | N/A | Survey | N/A | Takusa, Dembia and Libokemkem | Abera et al., 2022 |
| Tefluthrin |  | Rice, Maize | Survey | N/A | Takusa and Gondar zuria | Abera et al., 2022 |
| Acetamiprid |  | Cotton | Survey | N/A | Mecha, Bahir Dar Zuria, Fogera, Libokemkem | Tassew et al., 2023 |
| Clothianidin |  | Rice | Survey | N/A | Dera and Gondar zuria | Abera et al., 2022 |
| Imidacloprid |  | Potato and Tomato | Fish | Hi risk | Mecha, Bahir Dar Zuria, Fogera, Libokemkem,  Dera and Gondar zuria | Abera et al., 2022 |
| Thiamethoxam |  | Maize | Survey | N/A | Fogera | Abera et al., 2022 |
| Indoxacarb |  | Maize | Survey | N/A | Fogera | Abaineh et al., 2024 |
| Spiromesifen |  | N/A | Survey | N/A | Libokemkem | Abera et al., 2022 |
| Flubendiamide |  | N/A | Survey | N/A | Takusa and Libokemkem | Abera et al., 2022 |
| Abamectin |  | N/A | Survey | N/A | Alefa and Dembia | Abera et al., 2022 |
| Tebufenozide |  | N/A | Survey | N/A | Dembia and Gondar zuria | Abera et al., 2022 |
| Phenthoate |  | Tef, millet, wheat, barley | Survey | NA | Mecha, Bahir Dar Zuria, Fogera, Libokemkem | Tassew et al., 2023 |
| Novaluron |  |  | Survey | N/A | Takusa and Libokemkem | Abera et al., 2022 |
| Fenoxycarb |  | N/A | Survey | N/A | Libokemkem and Gondar zuria | Abera et al., 2022 |
| Azadirachtin |  | Tomato, Cabbage and Onion | Survey | N/A | Mecha, Bahir Dar Zuria, Fogera, Libokemkem | Tassew et al., 2023 |
| Lindane |  | Historically used | Fish, water and Sediment | High risk | Fogera, Bahir dar zuria, Gondar zuria ,and Libokemkem | Tibebe et al, 2022 |
| Endrin |  | N/A | Fish, water and Sediment | High risk | Fogera, Bahir dar zuria, Gondar zuria ,and Libokemkem | Tibebe et al, 2022 |
| Dieldrin |  | N/A | Fish, water and Sediment | High risk | Fogera, Bahir dar zuria, Gondar zuria ,and Libokemkem | Tibebe et al, 2022 |
| Oxamyl |  | N/A | Fish | High risk | Fogera and Libokemkem | Zelalem et al., 2023 |
| Cypermethrin |  | N/A | Water | No risk | Bahir dar zuria | Abera et al., 2022 |
| methomyl |  | N/A | Survey | N/A | Alefa and Libokemkem | Abera et al., 2022 |
| Propiconazole | Fungicides | Rice | Survey | N/A | Fogera | Abaineh et al., 2024 |
| Tebuconazole |  | Rice | Water | No risk | Fogera and Libokemkem | Zelalem et al., 2023 |
| Triadimefon |  | Wheat, Tomato, Cabbage , Onion  And Barley | Survey | N/A | Mecha, Bahir Dar Zuria, Fogera, Libokemkem | Tassew et al., 2023 |
| Triticonazole |  | N/A | Survey | N/A | Gondar Zuria | Abera et al., 2022 |
| Mancozeb |  | Maize, Vetch, Garlic ,Potato, Tomato, Onion, Pepper, Chick  pea, Cabbage , Lettuce and Tobacco | Survey | N/A | Fogera, Libokemkem,  Gondar Zuria, Mecha,  , Bahir Dar Zuria | Tassew et al., 2023; Abaineh et al., 2024 |
| Chlorothalonil |  | N/A | Water | No risk | South  Achefer, Alefa, Takusa,  Dembia,  Dera , Libokemkem and  Gonder  Zuria | Abera et al., 2022 |
| Metalaxyl |  | Garlic, Rice, Maize, Tomato, Onion, Pepper, Lettuce and  Cabbage | Survey | N/A | Fogera, Libokemkem,  Gondar Zuria, Mecha,  , Bahir Dar Zuria | Tassew et al., 2023; Abaineh et al., 2024 |
| Pyrimethanil |  | Rice | Fish | Low to moderate risk | Fogera and Libokemkem | Zelalem et al., 2023 |
| Difenoconazole |  | Wheat | Fish | No risk | Fogera and Libokemkem | Zelalem et al., 2023 |
| Benalaxyl |  | Rice | Fish | No risk | Fogera and Libokemkem | Zelalem et al., 2023 |
| 2,4-D | Herbicides | Millet, Mize, Sorghum,  Wheat, Teff, Barley and Rice | Survey | N/A | Fogera, Libokemkem,  Gondar Zuria, Mecha,  , Bahir Dar Zuria | Tassew et al., 2023; Abaineh et al., 2024 |
| 2,4-D amine |  | Vetch, Millet, Maize, Sorghum,  Wheat, Teff, Barley and Rice | Survey | N/A | Fogera, Libokemkem,  Gondar Zuria, Mecha,  , Bahir Dar Zuria | Tassew et al., 2023; Abaineh et al., 2024 |
| Atrazine |  | Vetch, Wheat, Teff and Barley | Survey | N/A | Fogera, Libokemkem,  Gondar Zuria, Mecha,  , Bahir Dar Zuria | Tassew et al., 2023; Abaineh et al., 2024 |
| Glyphosate |  | Millet, Maize, Sorghum | Survey | N/A | Bahir  Dar  Zuria,  North  Achefer,  South  Achefer, Mecha, Fogera, Libokemkem and  Gondar Zuria | Abera et al., 2022; Tassew et al., 2023 |
| Glyphosate-isopropylammonium |  | Millet, Maize, Sorghum,  Wheat, Teff and Barley | Survey | N/A | Mecha, Bahir Dar Zuria, Fogera, Libokemkem and  Gondar Zuria | Tassew et al., 2023 |
| Alachlor |  | N/A | Water | No risk | Dembia and Gondar zuria | Abera et al., 2022 |
| Butachlor |  | N/A | Fish | No risk | Fogera and Libokemkem | Zelalem et al., 2023 |
| Pyroxsulam |  | N/A | Survey | N/A |  |  |
| Flazasulfuron |  | N/A | Fish | High risk | Fogera and Libokemkem | Zelalem et al., 2023 |
| Zinc phosphide | Rodenticides | Rodents | Survey | N/A | Fogera, Libokemkem , Gorgora and Dera | Agmas and Adugna, 2020 |

Conceptual synthesis linking active pesticide ingredients reported in the Lake Tana sub-basin to their main crop uses, environmental detection locations, and reported ecological risk levels. Information on pesticide use was compiled from survey studies conducted across different districts of the basin (Agmas and Adugna, 2020; Abera et al., 2022; Tassew et al., 2023; Abaineh et al., 2024), while environmental occurrence and ecological risk characterisation were derived from monitoring studies reporting pesticide detections in water, sediment, and fish tissues (Tibebe et al., 2022; Zelalem et al., 2023). Ecological risk levels are reported only for pesticides detected in environmental matrices and for which risk quotient (RQ) assessments were available in the reviewed literature. “N/A” indicates that ecological risk assessment was not conducted because the pesticide was reported only through farmer surveys and not detected in environmental samples, or because insufficient data were available for RQ calculation**.**

**Table S4.** Summary of pesticides and the risks of their body residues in fish in the Lake Tana sub-basin.

| Sampling Sites | Pesticide | MEC  (µg/kg) | Lower detection limit (LOD, µg/kg) | PNEC  (µg/kg) | RQ value | Risk level | Analyzed tissue | Types of fish | References |
| --- | --- | --- | --- | --- | --- | --- | --- | --- | --- |
| Gumara | Carbaryl | 0.2 | 0.0003 | 115 | 0.0 | No risk | Muscle and liver | *Labeobarbus megastoma* | *Zelalem et al. 2023* |
|  | Flazasulfuron | 2.2 | 0.0003 | 0.04 | 55 | High risk | *Muscle and liver* |  |  |
|  | Oxamyl | 362 | 0.0003 | 3.36 | 108 | Very high risk | Muscle and liver |  |  |
|  | Pyrimethanil | 82.6 | 0.0003 | 38.7 | 2.1 | Low risk | Muscle and liver |  |  |
|  | Carbaryl | 0.4 | 0.0003 | 115 | 0.0 | No risk | Muscle and liver | *Labeobarbus tsanensis* | Zelalem et al. 2023 |
|  | Flazasulfuron | 2.2 | 0.0003 | 0.04 | 55 | High risk | Muscle and liver |  |  |
|  | Pyrimethanil | 30.1 | 0.0003 | 38.7 | 0.8 | No risk | Muscle and liver |  |  |
|  | Carbaryl | 4.6 | 0.0003 | 115 | 0.04 | No risk | Muscle and liver | *Oreochromis niloticus* | Zelalem et al. 2023 |
|  | Flazasulfuron | 2.2 | 0.0003 | 0.04 | 55 | High risk | Muscle and liver |  |  |
|  | Pyrimethanil | 55.1 | 0.0003 | 38.7 | 1.42 | Low risk | Muscle and liver |  |  |
|  | Benalaxyl | <LOD | 0.0003 | 1363 | 0.00000022 | No risk | Muscle and liver | *L. megastoma,* *L. tsanensis* and *O. niloticus* | Zelalem et al. 2023 |
|  | Butachlor | <LOD | 0.0003 | 36.8 | 0.00000815 | No risk | Muscle and liver |  |  |
|  | Difenoconazole | <LOD | 0.0003 | 443 | 0.00000068 | No risk | Muscle and liver |  |  |
|  | Pirimiphos-methyl | <LOD | 0.0003 | 0.071 | 0.00422535 | No risk | Muscle and liver |  |  |
|  | Tebuconazole | <LOD | 0.0003 | 9.31 | 0.00003222 | No risk | Muscle and liver |  |  |
| Ribb | Carbaryl | 0.4 | 0.0003 | 115 | 0.0 | No risk | Muscle and liver | *Labeobarbus megastoma* | Zelalem et al. 2023 |
|  | Flazasulfuron | 0.2 | 0.0003 | 0.04 | 4.7 | Low risk | Muscle and liver |  |  |
|  | Imidacloprid | 0.4 | 0.0003 | 0.013 | 31 | High risk | Muscle and liver |  |  |
|  | Pyrimethanil | 22.8 | 0.0003 | 38.7 | 0.6 | No risk | Muscle and liver |  |  |
|  | Flazasulfuron | 2.2 | 0.0003 | 0.04 | 55 | High risk | Muscle and liver | *Labeobarbus tsanensis* | Zelalem et al. 2023 |
|  | Imidacloprid | 2.2 | 0.0003 | 0.013 | 169 | Very high risk | Muscle and liver |  |  |
|  | Oxamyl | 24.4 | 0.0003 | 3.36 | 7.3 | Low risk | Muscle and liver |  |  |
|  | Pyrimethanil | 2.2 | 0.0003 | 38.7 | 0.1 | No risk | Muscle and liver |  |  |
|  | Carbaryl | 1.3 | 0.0003 | 115 | 0.0 | No risk | Muscle and liver | *Oreochromis niloticus* | Zelalem et al. 2023 |
|  | Flazasulfuron | 2.2 | 0.0003 | 0.04 | 55 | High risk | Muscle and liver |  |  |
|  | Imidacloprid | 0.4 | 0.0003 | 0.013 | 17 | High risk | Muscle and liver |  |  |
|  | Pyrimethanil | 2.2 | 0.0003 | 38.7 | 0.0 | No risks | Muscle and liver |  |  |
|  | Benalaxyl | <LOD | 0.0003 | 1363 | 0.00000022 | No risk | Muscle and liver | *L. megastoma, L. tsanensis* and *O. niloticus* | Zelalem et al. 2023 |
|  | Butachlor | <LOD | 0.0003 | 36.8 | 0.00000815 | No risk | Muscle and liver |  |  |
|  | Difenoconazole | <LOD | 0.0003 | 443 | 0.00000068 | No risk | Muscle and liver |  |  |
|  | Pirimiphos-methyl | <LOD | 0.0003 | 0.071 | 0.00422535 | No risk | Muscle and liver |  |  |
|  | Tebuconazole | <LOD | 0.0003 | 9.31 | 0.00003222 | No risk | Muscle and liver |  |  |
| Lake Tana | Lindane | 16.2 | 10 | 0.033 | 489 | Very high risk | Muscle | *Oreochromis niloticus* | Tibebe, 2022 |
|  | Endosulfan | 66.1 | 10 | 0.01 | 6610 | Very high risks | Muscle |  |  |
|  | DDT | 0.48 | 10 | 0.005 | 95 | High risks | Muscle |  |  |
|  | Endrin | 22.1 | 10 | 0.01 | 2212 | Very high risks | Muscle |  |  |
|  | Dieldrin | 11.2 | 10 | 0.2 | 56 | High risks | Muscle |  |  |

Note: PNECs are obtained from the NORMAN Ecotoxicology Database (<https://www.norman-network.com/nds/ecotox/>). Risk level characterization categories were established based on RQ values, with RQ < 1 indicating no risk, 1 to 10 low risk, 10 to 100 high risk, and RQ > 100 signifying very high risk (Merga et al. 2021). Values shown as “<LOD” indicate concentrations below the limit of detection. The LOD and LOQ for fish species (*Labeobarbus megastoma*, *Labeobarbus tsanensis* and *Oreochromis niloticus*) from the Gumara and Ribb rivers were 0.0003 μg/kg and 0.00086 μg/kg, respectively (Zelalem et al. 2023). For fish from Lake Tana, the LOD was 10 μg/kg (Tibebe 2022).

**Table S5.** Non-carcinogenic and carcinogenic human health risk assessment of pesticide residues in fish.

| **Carcinogenic and non-carcinogenic pesticide /WHO IARC carcinogenicity classification of pesticide)** | | | | | | | | | | |
| --- | --- | --- | --- | --- | --- | --- | --- | --- | --- | --- |
| Sampling Sites | Pesticide | Types of fish | MEC (mg/kg wet weight) | IR (kg/person/day) | BW (kg body weight) | EDI (mg/kg bw/day) | RfD / ADI (mg/kg bw/day) | Target Hazard Quotient (THQ) (-) | Risk level | Sources of (RfD) |
| Gumara and Ribb | Carbaryl | Labeobarbus megastoma | 0.0002 | 0.03 | 60 | 0.0000001 | 0.1 | 0.000001 | Acceptable | IRIS EPA |
|  | Difenoconazole |  | 0.0002 | 0.03 | 60 | 0.0000001 | 0.01 | 0.00001 | Acceptable | Alba Brancato et al., 2017 / EFSA |
|  | Imidacloprid |  | 0.0002 | 0.03 | 60 | 0.0000001 | 0.06 | 1.66667E-06 | Acceptable | Sebastian et al., 2021 |
|  | Oxamyl |  | 0.032 | 0.03 | 60 | 0.000016 | 0.025 | 0.00064 | Acceptable | IRIS EPA |
|  | Pyrimethanil |  | 0.0541 | 0.03 | 60 | 0.00002705 | 0.17 | 0.000159118 | Acceptable | Sebastian et al., 2021 |
|  | Teboconazole |  | 0.0002 | 0.03 | 60 | 0.0000001 | 0.03 | 3.33333E-06 | Acceptable | Sebastian et al., 2021 |
|  | Carbaryl | Labeobarbus tsanensis | 0.0004 | 0.03 | 60 | 0.0000002 | 0.1 | 0.000002 | Acceptable | IRIS EPA |
|  | Pyrimethanil |  | 0.0356 | 0.03 | 60 | 0.0000178 | 0.17 | 0.000104706 | Acceptable | Sebastian et al., 2021 |
| Lake Tana | Carbaryl | O. niloticus | 0.0031 | 0.03 | 60 | 0.00000155 | 0.1 | 0.0000155 | Acceptable | IRIS EPA |
|  | Imidacloprid |  | 0.0004 | 0.03 | 60 | 0.0000002 | 0.06 | 3.33333E-06 | Acceptable | Sebastian et al., 2021 |
|  | Pirimipho-methyl |  | 0.0004 | 0.03 | 60 | 0.0000002 | 0.03 | 6.66667E-06 | Acceptable | FAO/WHO (older JMPR) / EU |
|  | Pyrimethanil |  | 0.0949 | 0.03 | 60 | 0.00004745 | 0.17 | 0.000279118 | Acceptable | Sebastian et al., 2021 |
|  | Lindane |  | 0.01263 | 0.03 | 60 | 0.000006315 | 0.005 | 0.001263 | Acceptable | FAO/WHO (older JMPR) / EU |
|  | Endosulfan |  | 0.06878 | 0.03 | 60 | 0.00003439 | 0.006 | 0.005731667 | Acceptable | https://iris.epa.gov/AtoZ/ |
|  | DDT |  | 0.000516 | 0.03 | 60 | 0.000000258 | 0.0005 | 0.000516 | Acceptable | IRIS EPA |
|  | Endrin |  | 0.02177 | 0.03 | 60 | 0.000010885 | 0.0003 | 0.036283333 | Acceptable | IRIS EPA |
|  | Dieldrin |  | 0.01269 | 0.03 | 60 | 0.000006345 | 0.00005 | 0.1269 | Acceptable but relatively high | IRIS EPA |
| **For carcinogenic pesticide (US EPA/WHO IARC carcinogenicity classification of pesticide)** | | | | | | | | | | |
| Sampling sites Pesticides | Pesticide | Types of fish | MEC (mg/kg wet weight) | IR (kg/person/day) | BW (kg body weight) | EDI (mg/kg bw/day) | CSF (mg/kg-day)−1 | ILCR (-) | Risk level | Source |
| Lake Tana | Lindane | O. niloticus | 0.01263 | 0.03 | 60 | 0.000006315 | 1.1 | 6.9465E-06 | Negligible cancer concern | Li, 2018 |
|  | DDT |  | 0.000516 | 0.03 | 60 | 0.000000258 | 0.34 | 8.772E-08 | Low risk |  |
|  | Dieldrin |  | 0.01269 | 0.03 | 60 | 0.000006345 | 16 | 0.00010152 | High priority concern |  |

Noted: Estimated non-carcinogenic (Target Hazard Quotient, THQ) and carcinogenic (Incremental Lifetime Cancer Risk, ILCR) human health risks associated with consumption of fish contaminated with pesticide residues. Non-carcinogenic risk was assessed using estimated daily intake (EDI) and reference dose (RfD/ADI), while carcinogenic risk was evaluated using cancer slope factors (CSF) following US EPA and WHO/IARC guidelines. Risk levels were categorized according to internationally accepted thresholds. MEC refers to measured environmental concentration in fish tissue (wet weight). RfD and CSF values were obtained from US EPA IRIS, FAO/WHO (JMPR), EFSA, and published literature (Li, 2018; Sebastian et al., 2021)

**Table S6.** Summary of pesticide water residues and risk assessment results in the Lake Tana Sub-basin, expressed as PAF and msPAF (fraction) for each pesticide or mixture and sampling site

| Chemical | Sample | Concentration (µg/L) | PAFacute | PAFchronic | msPAFacute | msPAFchronic | Sample description | | References |
| --- | --- | --- | --- | --- | --- | --- | --- | --- | --- |
| p,p'-DDE | dL_AC_2:1-9-2019 | 0.11 | 0.01 | 0.10 | 0.01 | 0.10 | Shoreline near Achera wetland, associated with Lake Tana | Overgrown with invasive water hyacinth, which potentially affects pesticide accumulation | Abera et al. 2022 |
| Bifenthrin | dL_AC_2:1-9-2019 | <LOQ |  |  |  |  |  |  |  |
| DDT | dL_B/dar:1-5-2021 | 3.4 | 0.21 | 0.55 | 0.79 | 0.97 | Lake Tana shoreline Bahir Dar city | Area near Bahir Dar. City is a potential source of pollution from residential and urban activities | Tibebe 2022 |
| Endosulfan | dL_B/dar:1-5-2021 | 15 | 0.57 | 0.72 |  |  |  |  |  |
| Endrin | dL_B/dar:1-5-2021 | 1.2 | 0.34 | 0.73 |  |  |  |  |  |
| Lindane | dL_B/dar:1-5-2021 | 1.1 | 0.03 | 0.10 |  |  |  |  |  |
| DDT | dL_Deke:1-5-2021 | 3.7 | 0.22 | 0.57 | 0.79 | 0.98 | Lake Tana (open water) | Unobstructed, deeper parts of the lake |  |
| Dieldrin | dL_Deke:1-5-2021 | 1.1 | 0.10 | 0.37 |  |  |  |  |  |
| Endosulfan | dL_Deke:1-5-2021 | 12 | 0.54 | 0.69 |  |  |  |  |  |
| Endrin | dL_Deke:1-5-2021 | 1.1 | 0.34 | 0.72 |  |  |  |  |  |
| Lindane | dL_Deke:1-5-2021 | 1.1 | 0.03 | 0.10 |  |  |  |  |  |
| p,p'-DDE | dL_GO_2:1-9-2019 | 0.072 | 0.01 | 0.07 | 0.01 | 0.07 | Godeguadite wetland area, near Delgi town, on the Lake Tana shoreline | A lakeshore site impacted by overgrazing | Abera et al. 2022 |
| Bifenthrin | dL_GO_2:1-9-2019 | <LOQ |  |  |  |  |  |  |  |
| DDT | dL_Gorgora:1-5-2021 | 4.0 | 0.23 | 0.58 | 0.81 | 0.98 | Lake Tana shoreline | Near Gorgora resort, with potential wastewater input from the resort | Tibebe 2022 |
| Dieldrin | dL_Gorgora:1-5-2021 | 1.3 | 0.11 | 0.39 |  |  |  |  |  |
| Endosulfan | dL_Gorgora:1-5-2021 | 13 | 0.55 | 0.70 |  |  |  |  |  |
| Endrin | dL_Gorgora:1-5-2021 | 1.2 | 0.35 | 0.73 |  |  |  |  |  |
| Lindane | dL_Gorgora:1-5-2021 | 1.1 | 0.03 | 0.10 |  |  |  |  |  |
| p,p'-DDE | dL_LT2:1-9-2019 | 0.088 | 0.01 | 0.09 | 0.01 | 0.09 | Lake Tana, location 2 – Moonlight hotel/Bahir Dar city) | A potential source of pollution is wastewater discharge from the hotel | Abera et al. 2022 |
| Cypermethrin | dL_LT3:1-9-2019 | 0.55 | 0.45 | 0.44 | 0.45 | 0.44 | Lake Tana location 3 – Near St. George church | A potential source of pollution is wastewater discharge from the hotel |  |
| p,p'-DDE | dL_LT3:1-9-2019 | <LOQ |  |  |  |  |  |  |  |
| p,p'-DDE | dL_LT4:1-9-2019 | 0.098 | 0.01 | 0.09 | 0.01 | 0.09 | Lake Tana, location 4 – Fish production centre | Possible contributors to pollution in the gulf area near Bahir Dar City |  |
| Bifenthrin | dL_LT4:1-9-2019 | <LOQ |  |  |  |  |  |  |  |
| p,p'-DDE | dL_LT8:1-9-2019 | 0.082 | 0.01 | 0.08 | 0.01 | 0.08 | Lake Tana, location 8 – gulf of Delgi town | Possible sources of pollution in the northern gulf |  |
| Bifenthrin | dL_LT8:1-9-2019 | <LOQ |  |  |  |  |  |  |  |
| Bifenthrin | wL_LT8:1-9-2019 | <LOQ |  |  |  |  |  |  |  |
| Chlorothalonil | dL_LT8:1-9-2019 | <LOQ |  |  |  |  |  |  |  |
| DDT | dL_Sekelet:1-5-2021 | 3.4 | 0.21 | 0.55 | 0.80 | 0.98 | Located near the shore of Lake Tana | Intensive agricultural land use | Tibebe 2022 |
| Dieldrin | dL_Sekelet:1-5-2021 | 1.1 | 0.10 | 0.37 |  |  |  |  |  |
| Endosulfan | dL_Sekelet:1-5-2021 | 13 | 0.55 | 0.70 |  |  |  |  |  |
| Endrin | dL_Sekelet:1-5-2021 | 1.1 | 0.34 | 0.72 |  |  |  |  |  |
| Lindane | dL_Sekelet:1-5-2021 | 1.1 | 0.03 | 0.10 |  |  |  |  |  |
| DDT | dR_Gumara:1-5-2021 | 4.7 | 0.25 | 0.60 | 0.82 | 0.99 | Gumara river | Midstream of the river |  |
| Dieldrin | dR_Gumara:1-5-2021 | 1.5 | 0.12 | 0.42 |  |  |  |  |  |
| Endosulfan | dR_Gumara:1-5-2021 | 14 | 0.56 | 0.72 |  |  |  |  |  |
| Endrin | dR_Gumara:1-5-2021 | 1.4 | 0.37 | 0.75 |  |  |  |  |  |
| Lindane | dR_Gumara:1-5-2021 | 1.1 | 0.03 | 0.10 |  |  |  |  |  |
| DDT | dR_Megech:1-5-2021 | 4.7 | 0.25 | 0.60 | 0.83 | 0.99 | Megech river | River Mouth area |  |
| Dieldrin | dR_Megech:1-5-2021 | 1.2 | 0.10 | 0.37 |  |  |  |  |  |
| Endosulfan | dR_Megech:1-5-2021 | 17 | 0.59 | 0.74 |  |  |  |  |  |
| Endrin | dR_Megech:1-5-2021 | 1.3 | 0.36 | 0.74 |  |  |  |  |  |
| Lindane | dR_Megech:1-5-2021 | 1.1 | 0.03 | 0.10 |  |  |  |  |  |
| p,p'-DDE | dR_RD_2:1-9-2019 | 0.060 | 0.01 | 0.06 | 0.01 | 0.06 | Dirma River, Location 2 – river mouth area | Site in proximity to a large-scale crop cultivation area | Abera et al. 2022 |
| p,p'-DDE | dR_RE_1:1-9-2019 | 0.12 | 0.02 | 0.11 | 0.02 | 0.11 | Enfranze river, location 1 – upstream | Located near an urban area and exposed to contamination from laundry detergents |  |
| Bifenthrin | dR_RE_1:1-9-2019 | <LOQ |  |  |  |  |  |  |  |
| p,p'-DDE | dR_RE_2:1-9-2019 | 0.089 | 0.01 | 0.09 | 0.01 | 0.09 | Enfranze river, location 2 – river mouth | Covered with macrophytes and exposed to potential diffuse pollution sources |  |
| Bifenthrin | dR_RE_2:1-9-2019 | <LOQ |  |  |  |  |  |  |  |
| p,p'-DDE | dR_RG_1:1-9-2019 | 0.11 | 0.02 | 0.10 | 0.02 | 0.10 | Gumara river, location 1 – upstream | Vegetation present along the riverbanks |  |
| Bifenthrin | dR_RG_1:1-9-2019 | <LOQ |  |  |  |  |  |  |  |
| p,p'-DDE | dR_RG_2:1-9-2019 | 0.089 | 0.01 | 0.09 | 0.01 | 0.09 | Gumara river location 2 – river mouth | A site located near potential sources of diffuse pollution |  |
| p,p'-DDE | dR_RGA_1:1-9-2019 | 0.096 | 0.01 | 0.09 | 0.01 | 0.09 | Gilgel Abay river location 1 – upstream | Non-point pollution source from nearby agriculture, including onion and maize cultivation |  |
| Chlorothalonil | wR_RGA_1:1-9-2019 | <LOQ |  |  |  |  |  |  |  |
| Bifenthrin | wR_RGA_1:1-9-2019 | <LOQ |  |  |  |  |  |  |  |
| Bifenthrin | dR_RGA_1:1-9-2019 | <LOQ |  |  |  |  |  |  |  |
| p,p'-DDE | dR_RGA_2:1-9-2019 | 0.080 | 0.01 | 0.08 | 0.01 | 0.08 | Gilgel Abay river, location 2 – river mouth | At the river mouth floodplain, a site affected by cattle overgrazing |  |
| Bifenthrin | wR_RGA_2:1-9-2019 | <LOQ |  |  |  |  |  |  |  |
| Bifenthrin | dR_RGA_2:1-9-2019 | <LOQ |  |  |  |  |  |  |  |
| DDT | dR_Ribb:1-5-2021 | 5.0 | 0.26 | 0.61 | 0.86 | 0.99 | Ribb river | Midstream of the river | Tibebe 2022 |
| Dieldrin | dR_Ribb:1-5-2021 | 2.5 | 0.17 | 0.51 |  |  |  |  |  |
| Endosulfan | dR_Ribb:1-5-2021 | 19 | 0.60 | 0.76 |  |  |  |  |  |
| Endrin | dR_Ribb:1-5-2021 | 1.8 | 0.42 | 0.79 |  |  |  |  |  |
| Lindane | dR_Ribb:1-5-2021 | 1.2 | 0.03 | 0.10 |  |  |  |  |  |
| O,P'-DDT | dR_RR_1:1-9-2019 | 0.32 |  |  |  |  | Ribb river location 1 – upstream | Located near an urban area and exposed to contamination from laundry detergents | Abera et al. 2022 |
| Bifenthrin | dR_RR_1:1-9-2019 | <LOQ |  |  |  |  | Ribb river location 1 – upstream | Located near an urban area and exposed to contamination from laundry detergents |  |
| p,p'-DDE | dR_RR_1:1-9-2019 | 0.11 | 0.01 | 0.10 | 0.01 | 0.10 |  |  |  |
| O,P'-DDT | dW_GI_1:1-9-2019 | 0.17 |  |  |  |  | Gilgel Abay wetland is connected to the lake | Macrophyte presence, including species like papyrus |  |
| Bifenthrin | dW_GI_1:1-9-2019 | <LOQ |  |  |  |  |  |  |  |
| p,p'-DDE | dW_LM_2:1-9-2019 | 0.094 | 0.01 | 0.09 | 0.01 | 0.09 | Lemba wetland is connected to the lake | Wetland site exposed to water hyacinth |  |
| Bifenthrin | dW_LM_2:1-9-2019 | <LOQ |  |  |  |  |  |  |  |
| Alachlor | dW_ME_1:1-9-2019 | 1.1 | 0.00 | 0.00 | 0.00 | 0.00 | Megech Wetland, connected to the lake | Grazing and farming are commonly practiced |  |
| Bifenthrin | dW_ME_1:1-9-2019 | <LOQ |  |  |  |  |  |  |  |
| p,p'-DDE | dW_MW_3:1-9-2019 | 0.092 | 0.01 | 0.09 | 0.01 | 0.09 | MeTsri-Abawarka wetland, connected to the lake | A wetland site affected by water hyacinth |  |
| Bifenthrin | dW_MW_3:1-9-2019 | <LOQ |  |  |  |  |  |  |  |
| Alachlor | dW_RDG:1-9-2019 | 0.29 | 0.00 | 0.00 | 0.00 | 0.00 | Dehena Mesenta wetland | Exposed to an agricultural site and grazing in the western part of the lake |  |
| p,p'-DDE | dW_RDG:1-9-2019 | <LOQ |  |  |  |  |  |  |  |
| Alachlor | dW_RDU:1-9-2019 | 0.36 | 0.00 | 0.00 | 0.00 | 0.00 | Dehena Mesenta wetland | Minimal grazing with good vegetation cover, such as papyrus |  |
| p,p'-DDE | dW_RDU:1-9-2019 | <LOQ |  |  |  |  |  |  |  |
| Bifenthrin | dW_RDU:1-9-2019 | <LOQ |  |  |  |  |  |  |  |
| p,p'-DDE | wL_GO_2:1-9-2019 | 0.11 | 0.02 | 0.10 | 0.02 | 0.10 | Godeguadite wetland area, near Delgi town, on the lake’s shoreline | A lakeshore site impacted by overgrazing |  |
| Bifenthrin | wL_GO_2:1-9-2019 | <LOQ |  |  |  |  |  |  |  |
| p,p'-DDE | wL_LT6:1-9-2019 | 0.082 | 0.01 | 0.08 | 0.01 | 0.08 | Lake Tana, location 6 – Blue Nile River outflow | A potentially unimpacted site located in the southern part. |  |
| p,p'-DDE | dL_LT6:1-9-2019 | <LOQ |  |  |  |  |  |  |  |
| Bifenthrin | wL_LT6:1-9-2019 | <LOQ |  |  |  |  |  |  |  |
| p,p'-DDE | wL_LT8:1-9-2019 | 0.10 | 0.01 | 0.09 | 0.01 | 0.09 | Lake Tana, location 8 – gulf of Delgi town | Possible sources of pollution in the northern gulf |  |
| Cypermethrin | wL_ZI_2:1-9-2019 | 0.14 | 0.26 | 0.31 | 0.27 | 0.37 | Zegie-yiganda wetland, at lake shoreline | A relatively pristine site located near the Zegie- yiganda wetland |  |
| Bifenthrin | wL_ZI_2:1-9-2019 | <LOQ |  |  |  |  |  |  |  |
| p,p'-DDE | wL_ZI_2:1-9-2019 | 0.085 | 0.01 | 0.08 |  |  |  |  |  |
| p,p'-DDE | wR_RD_2:1-9-2019 | 0.11 | 0.02 | 0.10 | 0.02 | 0.10 | Dirma River, Location 2 – river mouth area | Site in proximity to a large-scale crop cultivation area |  |
| Bifenthrin | wR_RD_2:1-9-2019 | <LOQ |  |  |  |  |  |  |  |
| p,p'-DDE | wR_RE_1:1-9-2019 | 0.10 | 0.01 | 0.10 | 0.01 | 0.10 | Enfranze river, location 1 – upstream | Located near an urban area and exposed to contamination from laundry detergents |  |
| Bifenthrin | wR_RE_1:1-9-2019 | <LOQ |  |  |  |  |  |  |  |
| p,p'-DDE | wR_RE_2:1-9-2019 | 0.11 | 0.02 | 0.10 | 0.02 | 0.10 | Enfranze river, location 2 – river mouth | Covered with macrophytes and exposed to potential diffuse pollution sources |  |
| Bifenthrin | wR_RE_2:1-9-2019 | <LOQ |  |  |  |  |  |  |  |
| p,p'-DDE | wR_RG_1:1-9-2019 | 0.11 | 0.02 | 0.10 | 0.02 | 0.10 | Gumara river, location 1 – upstream | Vegetation present along the riverbanks |  |
| p,p'-DDE | wR_RG_2:1-9-2019 | 0.12 | 0.02 | 0.11 | 0.02 | 0.11 | Gumara river location 2 – river mouth | A site located near potential sources of diffuse pollution |  |
| Bifenthrin | wR_RG_2:1-9-2019 | <LOQ |  |  |  |  |  |  |  |
| Alachlor | wR_RG_2:1-9-2019 | <LOQ |  |  |  |  |  |  |  |
| p,p'-DDE | wR_RGA_1:1-9-2019 | 0.099 | 0.01 | 0.09 | 0.01 | 0.09 | Gilgel Abay river location 1 – upstream | Non-point pollution source from nearby agriculture, including onion and maize cultivation |  |
| Bifenthrin | wR_RGA_1:1-9-2019 | <LOQ |  |  |  |  |  |  |  |
| p,p'-DDE | wR_RGA_2:1-9-2019 | 0.11 | 0.01 | 0.10 | 0.01 | 0.10 | Gilgel Abay river, location 2 – river mouth | At the river mouth floodplain, a site affected by cattle overgrazing |  |
| p,p'-DDE | wR_RM_2:1-9-2019 | 0.10 | 0.01 | 0.10 | 0.01 | 0.10 | Megech river, Location 2 – river mouth at the northern part of the lake | Site located near a crop cultivation area and cattle grazing land |  |
| p,p'-DDE | dR_RM_2:1-9-2019 | <LOQ |  |  |  |  |  |  |  |
| p,p'-DDE | wR_RR_1:1-9-2019 | 0.080 | 0.01 | 0.08 | 0.01 | 0.08 | Ribb river location 1 upstream | Located near an urban area and exposed to contamination from laundry detergents |  |
| p,p'-DDE | wR_RR_2:1-9-2019 | 0.11 | 0.01 | 0.10 | 0.01 | 0.10 | Ribb river location 2 – river mouth | Presence of vegetation at riverbank and overall undisturbed |  |
| Bifenthrin | wR_RR_2:1-9-2019 | <LOQ |  |  |  |  |  |  |  |
| p,p'-DDE | dR_RR_2:1-9-2019 | <LOQ |  |  |  |  |  |  |  |

Note: PAF Acute = potentially affected fraction for acute exposure; PAF Chronic = potentially affected fraction for chronic exposure; msPAF = multi-substance potentially affected fraction; (“–”) indicates missing values where toxicity data were unavailable. Samples are grouped by location and seasons: “dL” = dry season lake samples; “dR” = dry season river samples; “dW” = dry season wetland samples; “wL” = wet season lake samples; and “wR” = wet season river samples. The numbers “1” and “2” indicate sampling locations within rivers, where “1” refers to upstream sites and “2” to river mouths. Values reported as “<LOQ” denote concentrations below the limit of quantification (LOQ). The limits of detection (LOD) and LOQ for the analysed pesticides ranged from 0.01 to 0.2 μg/L and from 0.04 to 0.5 μg/L, respectively.

**Table S7.** Measured concentrations and ecological risk characterization of pesticide residues in sediment.

| Sampling sites | Pesticide | MEC (μg/kg) | PNEC (µg/kg) | RQ value | Risk level | References |
| --- | --- | --- | --- | --- | --- | --- |
| Bahir Dar | Lindane | 10.12 | 0.0011 | 9200.00 | High risk | Tibebe et al.,2022 |
|  | Endosulfan | 33.32 | 3.19 | 10.45 | High risk |  |
|  | DDT | 0.496 | 0.01 | 49.60 | High risk |  |
|  | Endrin | 125.65 | 0.04 | 3141.25 | High risk |  |
|  | Dieldrin | 16.61 | 0.005 | 3322.00 | High risk |  |
| Gorgora | Lindane | 11.78 | 0.0011 | 10709.09 | High risk |  |
|  | Endosulfan | 28.22 | 3.19 | 8.85 | High risk |  |
|  | DDT | 0.432 | 0.01 | 43.20 | High risk |  |
|  | Endrin | 60.5 | 0.04 | 1512.50 | High risk |  |
|  | Dieldrin | 16.09 | 0.005 | 3218.00 | High risk |  |
| Sekelet | Lindane | 11.09 | 0.0011 | 10081.82 | High risk |  |
|  | Endosulfan | 23.09 | 3.19 | 7.24 | High risk |  |
|  | DDT | 0.46 | 0.01 | 46.00 | High risk |  |
|  | Endrin | 54.6 | 0.04 | 1365.00 | High risk |  |
|  | Dieldrin | 13.17 | 0.005 | 2634.00 | High risk |  |
| Deke | Lindane | 10.18 | 0.0011 | 9254.55 | High risk |  |
|  | Endosulfan | 23.99 | 3.19 | 7.52 | High risk |  |
|  | DDT | 0.497 | 0.01 | 49.70 | High risk |  |
|  | Endrin | 40.2 | 0.04 | 1005.00 | High risk |  |
|  | Dieldrin | 11.91 | 0.005 | 2382.00 | High risk |  |
| Gumara | Lindane | 11.85 | 0.0011 | 10772.73 | High risk |  |
|  | Endosulfan | 35.25 | 3.19 | 11.05 | High risk |  |
|  | DDT | 0.446 | 0.01 | 44.60 | High risk |  |
|  | Endrin | 56.8 | 0.04 | 1420.00 | High risk |  |
|  | Dieldrin | 15.64 | 0.005 | 3128.00 | High risk |  |
| Ribb | Lindane | 12.66 | 0.0011 | 11509.09 | High risk |  |
|  | Endosulfan | 37.67 | 3.19 | 11.81 | High risk |  |
|  | DDT | 0.439 | 0.01 | 43.90 | High risk |  |
|  | Endrin | 68.9 | 0.04 | 1722.50 | High risk |  |
|  | Dieldrin | 14.54 | 0.005 | 2908.00 | High risk |  |
| Megech | Lindane | 11.64 | 0.0011 | 10581.82 | High risk |  |
|  | Endosulfan | 33.42 | 3.19 | 10.48 | High risk |  |
|  | DDT | 0.377 | 0.01 | 37.70 | High risk |  |
|  | Endrin | 86.3 | 0.04 | 2157.50 | High risk |  |
|  | Dieldrin | 16.83 | 0.005 | 3366.00 | High risk |  |
| Fogera | DDT | 1.77 | 0.01 | 177.00 | High risk | Abaineh et al., 2025 |
|  | Endosulfan | 1.9 | 3.19 | 0.60 | Low risk |  |
|  | Endrin | 0.75 | 0.04 | 18.75 | High risk |  |

Measured environmental concentrations (MEC, μg/kg dry weight) of pesticide residues detected in sediment samples from different sampling sites (Tibebe et al.,2022; Abaineh et al., 2025), the predicted no-effect concentrations (PNEC) PNECs are obtained from the NORMAN Ecotoxicology Database (<https://www.norman-network.com/nds/ecotox/> to calculate risk quotient (RQ = MEC/PNEC). Risk characterization is based on standard thresholds, where RQ < 1 indicates low risk, 1 ≤ RQ < 10 indicates moderate risk, and RQ ≥ 10 indicates high ecological risk (Nie et al., 2015).
